# Phylogeography and phenotypic wing shape variation in a damselfly across populations in Europe

**DOI:** 10.1186/s12862-024-02207-4

**Published:** 2024-02-03

**Authors:** Y. Yildirim, D. Kristensson, D. Outomuro, D. Mikolajewski, P. Rödin Mörch, S. Sniegula, F. Johansson

**Affiliations:** 1 Department of Ecology and Genetics, Animal Ecology, Uppsala University, Uppsala, Sweden; 2 Department of Biological Sciences, University of Pittsburgh, Pittsburgh, PA USA; 3 Institut für Biologie, Freie Universität Berlin, Berlin, Germany; 4 Department of Ecosystem Conservation, Institute of Nature Conservation, Polish Academy of Sciences, Kraków, Poland

**Keywords:** Wing shape, Adaptation, Latitude, Population structure, Phylogeography, Damselfly

## Abstract

**Background:**

Describing geographical variation in morphology of organisms in combination with data on genetic differentiation and biogeography can provide important information on how natural selection shapes such variation. Here we study genetic structure using ddRAD seq and wing shape variation using geometric morphometrics in 14 populations of the damselfly *Lestes sponsa* along its latitudinal range in Europe.

**Results:**

The genetic analysis showed a significant, yet relatively weak population structure with high genetic heterozygosity and low inbreeding coefficients, indicating that neutral processes contributed very little to the observed wing shape differences. The genetic analysis also showed that some regions of the genome (about 10%) are putatively shaped by selection. The phylogenetic analysis showed that the Spanish and French populations were the ancestral ones with northern Swedish and Finnish populations being the most derived ones.

We found that wing shape differed significantly among populations and showed a significant quadratic (but weak) relationship with latitude. This latitudinal relationship was largely attributed to allometric effects of wing size, but non-allometric variation also explained a portion of this relationship.

However, wing shape showed no phylogenetic signal suggesting that lineage-specific variation did not contribute to the variation along the latitudinal gradient. In contrast, wing size, which is correlated with body size in *L. sponsa*, had a strong negative correlation with latitude.

**Conclusion:**

Our results suggest a relatively weak population structure among the sampled populations across Europe, but a clear differentiation between south and north populations. The observed geographic phenotypic variation in wing shape may have been affected by different local selection pressures or environmental effects.

**Supplementary Information:**

The online version contains supplementary material available at 10.1186/s12862-024-02207-4.

## Background

Spatial environmental heterogeneity is a key driver for organismal trait variation in physiology, morphology and behavior among populations [1, 2]. One challenge for evolutionary biologists is to link local selective constraints within a particular habitat to observed trait values. Well-studied examples for population mediated differences in selection pressures are variation in predation risk or sexual selection, as well as variation in temperature or humidity [3–5]. Understanding such variation among populations is not trivial because the effects of selection may be counterbalanced by phenotypic plasticity, genetic drift, migration and organism’s evolutionary history [2]. Thus, for a better understanding of the observed phenotypic variation of a species across its distribution range, analyses of trait values in relation to genetic population structure are needed.

Understanding current phenotypic variation across a species range can be facilitated with knowledge on the current genetic structure among its populations, and information about colonization history. Measures on genetic divergence may provide information on which populations are isolated from each other. Such isolation may shape different trait values among populations via distinctive local selection pressures [2, 6]. However, colonization history may also affect trait values via founder effects [7] or locally mediated adaptive selection along the colonization route [8, 9]. By mapping trait values such as morphology in a phylogeographical context, information about a species colonization history and potential adaptation across the geographical range of a species can be understood [10].

One spectacular trait that has been shown to vary among and within many species are wings. Most insect species have wings, and variation in wing shape within a species is an important component affecting fitness traits such as mating success [11], dispersal [12], and predator avoidance [13]. In addition, many species show variation in wing shape across their geographical distribution [12, 14–16]. Large parts of this variation have been attributed to temperature effects along latitudinal gradients [17, 18]. However, the majority of these studies are limited to *Drosophila* and other dipterans which have one active wing pair (reviewed in [18]). Besides temperature, biological factors such as differences in predation risk and sexual selection can affect wing shape [13, 19]. Key findings in a species of damselfly revealed selection was mediated by predation favoring a slender forewing shape and a short and broad hindwing shape [13]. Moreover, data in another damselfly species, *Lestes sponsa* (Hansemann), exposed to sexual selection favored short and broad forewings and narrow-based hindwings [19]. However, there is a lack of studies exploring the differences in wing shape in insects across a large geographical scale, particularly in combination with detailed data on genetic differentiation among populations and their invasion history. Such data provides valuable information on the presence or absence of general trends in wing shape along latitudinal gradients.

**Fig. 1:**
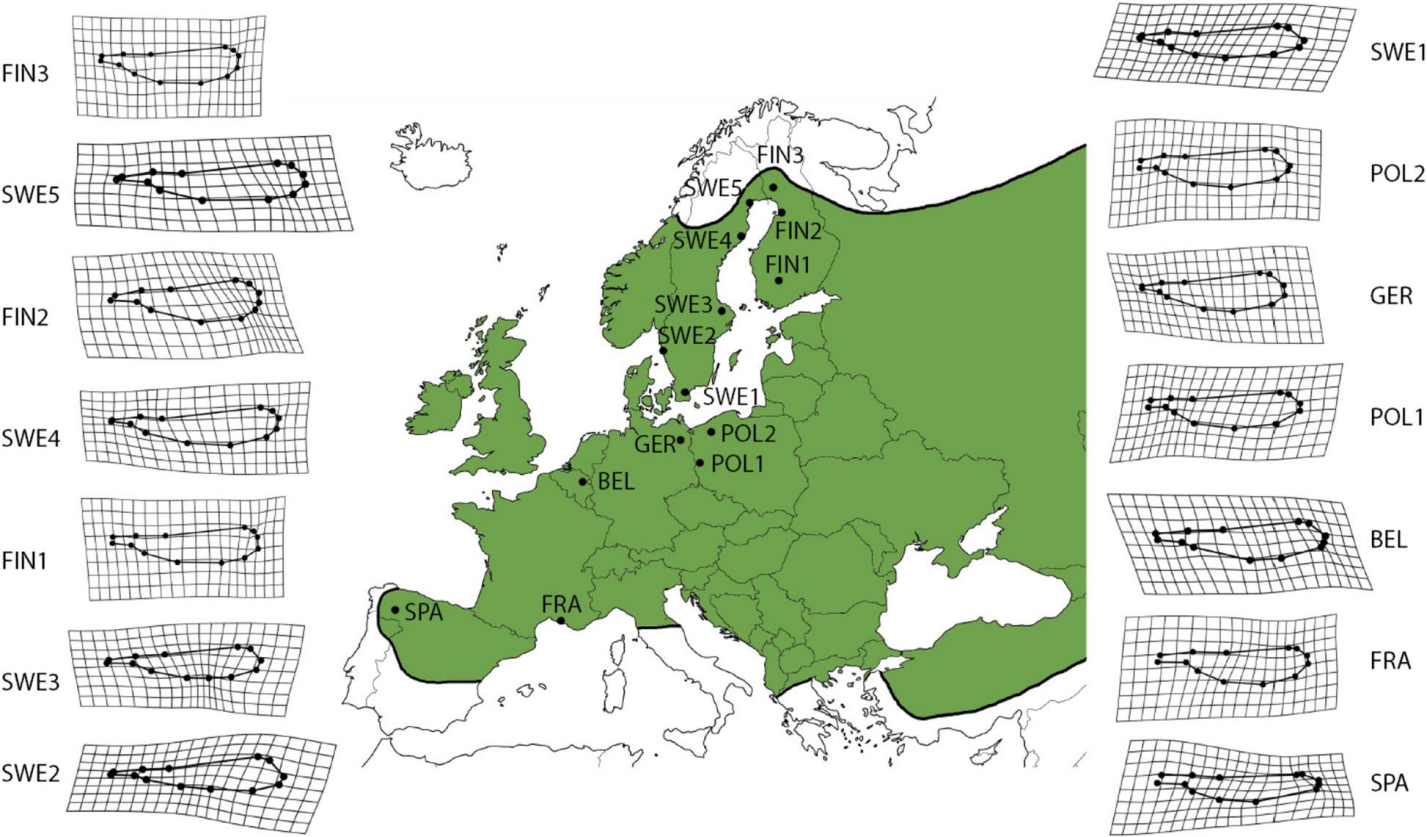
Distribution of *Lestes sponsa* in Europe (shaded area), and the 14 sampling locations. Forewing shape variation among populations is shown as deformation grids, and grids are enhanced ×10 times for ease of visualization (for hindwing shape variation, see Suppl. Fig. S6). Information for creating the map was taken from [20]

The damselfly *L. sponsa* is widespread throughout Europe except for the Mediterranean and very northern Fennoscandia [20] (Fig. 1). This species is well suited to study wing shape variation along a latitudinal gradient. Past studies have shown some evidence for local adaptation in populations along a latitudinal gradient. For example, there is local adaptive variation in wing shape [19] as well as genetic variation in wing shape and life history traits along the latitudinal gradient [21–23]. In addition, also body size varies across latitudes and one study has shown a U-shaped latitudinal pattern [16]. However, we lack detailed knowledge on how *L. sponsa* is genetically differentiated across a larger geographical area and on its phylogeography. Such information is needed if we want to confirm that wing shape variation is adaptive and not a consequence of genetic drift or founder effects. Past studies on other odonates have suggested glacial refugia in the south-west and south-east of Europe with subsequent dispersal routes to the north following western and eastern routes [24]. Such a dispersal route is probably also present in *L. sponsa* and has the potential to affect current patterns in wing shape along its latitudinal gradient. In damselflies, dispersal is probably favored by long and slender wings [25], but no such evidence has been shown for *L. sponsa* [16]. However, at the local scale, sexual selection favors short and broad forewings and narrow-based hindwings [19], while survival favors long and slender forewings and short and broad hindwings [19], which could counterbalance selection caused by dispersal. It is therefore interesting to further explore wing shape along a latitudinal gradient in *L. sponsa*. Finally, there are phenotypic differences in wing shape among *L. sponsa* populations across the latitudinal gradient in Europe, but we note that latitudinal effects account for a low percentage of the total wing shape variation across this gradient [16]. Since there was a low percentage of wing shape variation explained by the latitudinal gradient in *L. sponsa*, it would be interesting to explore if the absence of a strong latitudinal pattern in wing shape is also reflected in an absence of a strong genetic structure along the latitudinal gradient in *L. sponsa*. If there is a genetic structure along the latitudinal gradient this would suggest genetic drift or genetic adaptation in traits not necessarily related to wing shape. Here we use a completely new data set to examine how phenotypic wing shape of *L. sponsa* varies across a large geographic area in Europe and how this wing shape variation is associated with genetic structure and phylogeography. We explore the genetic structure with an outlier analysis using SNPs. There is no overlap with this new wing morphology data set and the old one and the new data set includes DNA sequencing data which allows us to analyze genetic structure. We predicted that: (1) wing shape should not show a phylogenetic signal, because we expect local adaptation in wing shape across the populations, not necessarily corresponding to genetic relationships; (2) a slender wing shape in northern populations at range margins because slender wings are beneficial for dispersal in damselflies [25]; (3) the phylogeny of the sampled populations should reflect dispersal from the glacial refuges so that the ancestral population should be from the southern populations and the northern populations should derive from it; and (4) if there is population differentiation this might have been driven by adaptive differentiation.

## Material and methods

### Study area and data collection

The study species *L. sponsa* has a one-year obligate life cycle, overwintering in the egg stage, and showing a synchronous hatching in the summer with a larval development of 2–3 months [26]. Adult males of *L. sponsa* were captured from 14 different locations along a latitudinal gradient in Europe from June to August in 2020 (Fig.1, Table 1). Our focus was on males because our past studies have found indication that male wing size is related to fitness components [19], and we wanted to explore how such wing shape variation varies with latitude. The sampling area covered 42.161824–66.278367° latitude, and − 6.974968 – 25.484802° longitude (Fig. 1). The damselflies were preserved in 96% alcohol upon being caught with a butterfly net, and thereafter sent to the Department of Ecology and Genetics at Uppsala University.

**Table 1:** Sampling locations and genetic diversity. Sample size (*N*) of forewings, hindwings, and genotyped individuals with ddRADseq from each sampling location. Variant sites for each population, private alleles (PA), observed (*H*_O_) and expected heterozygosity (*H*_E_), and fixation index (*F*_is_) for each population. SE: standard error

					N wings							
Populationnumber	Population name	Sampling location	Latitude	Longitude	forewing	hindwing	N genotyped	Variant sites	PA	HO (SE)	HE (SE)	Fis (SE)
1	SPA	Laguna de Lombo,Ourense, Spain	42.16182	−6.97497	10	13	3	3968	0	0.125 (0.002)	0.105 (0.001)	0.026 (0.006)
2	FRA	Arles, France	43.47511	4.81802	16	16	7	10,459	1	0.182 (0.002)	0.204 (0.001)	0.1 (0.008)
3	BEL	Oude Beek, Sint-Truiden, Belgium	50.83758	5.232083	14	17	5	10,417	0	0.204 (0.002)	0.212 (0.001)	0.076 (0.005)
4	POL1	Pław, Poland	51.99017	15.19383	12	14	7	11,419	0	0.203 (0.002)	0.212 (0.001)	0.068 (0.008)
5	GER	Boitzenburger Land, Germany	53.24808	13.58941	9	10	7	10,912	0	0.197 (0.002)	0.208 (0.001)	0.076 (0.01)
6	POL2	Czarne Wielkie, Poland	53.65767	16.27406	13	11	7	9285	0	0.221 (0.002)	0.218 (0.002)	0.056 (0.014)
7	SWE1	Sallerup, Hörby, Sweden	55.79888	13.73272	9	9	7	11,782	0	0.215 (0.002)	0.22 (0.001)	0.062 (0.007)
8	SWE2	Långekärr, Tjörn, Sweden	58.03273	11.56131	9	12	7	12,144	0	0.223 (0.002)	0.224 (0.001)	0.048 (0.005)
9	SWE3	Stenhagen, Uppsala, Sweden	59.84903	17.5493	10	10	7	11,740	0	0.218 (0.002)	0.221 (0.001)	0.053 (0.004)
10	FIN1	Katumajärvi, Hämeenlinna, Finland	60.99038	24.5168	11	12	7	12,202	0	0.222 (0.002)	0.223 (0.001)	0.049 (0.004)
11	SWE4	Hamptjärnen, Umeå, Sweden	63.76185	20.04773	7	11	7	11,972	0	0.217 (0.002)	0.222 (0.001)	0.058 (0.004)
12	FIN2	Pyykösjärvi, Oulu, Finland	65.048	25.492	11	12	7	11,877	0	0.208 (0.002)	0.222 (0.001)	0.081 (0.004)
13	SWE5	Mjölkuddstjärnen, Luleå, Sweden	65.60597	22.12855	10	13	7	8873	0	0.222 (0.002)	0.217 (0.002)	0.053 (0.015)
14	FIN3	Montosenlampi, Rovaniemi, Finland	66.27837	25.4848	12	13	7	11,458	0	0.217 (0.002)	0.219 (0.001)	0.052 (0.007)

### Library preparation and ddRAD-sequencing

To examine genetic structure and phylogeography, we randomly selected 7 *L. sponsa* individuals from each of the 14 sampled localities (in total *n* = 98) to prepare double digest restriction associated DNA sequencing (ddRAD) libraries. Genomic DNA was extracted by salting out method described by [27], with some modifications. The ddRAD libraries were created using a modified version of protocols from [28–30]. DNA was digested by the enzymes EcoRI-HF and MseI, individual barcodes and primer sites were ligated with T4 DNA ligase, and PCR was conducted with Q5 DNA polymerase (New England Biolabs, Massachusetts, USA). After PCR, the samples were pooled, and size selection was performed in agarose gel. The library was sequenced in a single lane on an Illumina Novaseq 6000 machine from both directions (2 × 150 bp) at SciLifeLab, Uppsala, Sweden.

### Data filtering and SNP calling

Trimming of adapters/primers and demultiplexing of the raw data were performed in CUTADAPT v4.0 [31]. All the reads were trimmed to a uniform length of 100 bp, and the reads with quality score < phred33 discarded in trimmomatic v 0.39 [32]. De novo pipeline in stacks v.2.52 [33] was used for SNP calling by running each step of the pipeline (*ustacks*, *cstacks*, *sstacks*, *tsv2bam* and *gstacks*) separately. A parameter search on 14 randomly selected individuals (one individual from each locality) was first performed as described in [34, 35] to identify the optimum setup for the parameters *m, M* and *n.* Parameter *m* is the minimum number of raw reads to form a stack or putative allele within an individual, *M* is the maximum number of mismatches allowed between stacks to form a putative locus, and *n* is the number of mismatches allowed between individual loci across samples to build a catalogue of loci across individuals. We decided to use 6 for all *m*, *M* and *n* parameters (Supplement: Figs. S1 and S2). We ran the de novo pipeline on 98 samples using these parameters and otherwise default settings. The resulting catalog contained 2,414,272 loci with a mean coverage of 25.0x (standard deviation = 10.2x) per sample. The catalog was further processed with the *populations* unit in stacks with R*80* setup, where a locus was retained if it was present in at least 80% of individuals. We also chose a minimum minor allele frequency of 0.05, a maximum observed heterozygosity of 0.7 and one random SNP per locus. The *populations* step was repeated after removing 6 samples with > 50% missing data (4 from Spain and 2 from Belgium). The final dataset contained 16,146 biallelic SNPs (Supplement: Table S1). This dataset was used for most of the further statistical analyses. A stricter filtering was applied whenever required by an analysis.

### Genetic structure

Multiple methods to study the genetic structure of the *L. sponsa* populations were used. Molecular diversity indices including observed heterozygosity (*H*_*O*_), expected heterozygosity (*H*_*E*_), and fixation index (*Fis*) were estimated for each population in the software STACKS. The violation of Hardy Weinberg equilibrium (HWE) was also tested in the same software.

Pairwise differentiation among the sampling locations was estimated using *F*_st_ [36] in Arlequin v3.5.5 with 1000 iterations (16,146 SNPs). Principal Component Analysis (PCA) through the package *pcadapt* [37] in R [38] was performed to visualize the population structure. A haplotype-based approach implemented in fineRADstructure [39] for a formal clustering analysis was used to evaluate shared ancestry among the individuals. STRUCTURE v.2.3.4 [40, 41] was used as a Bayesian approach to determine the most likely number of genetic clusters (*K*) by running the analysis for each number of *K* (1–12) with 10 iterations for each *K*. We used an admixture model introducing sampling location as a priori information and assuming correlated allele frequencies [41]. Lambda was set to 0.7009 after determined with a pre-run. STRUCTURESELECTOR [42] was used to assess the best *K* that explains the data using the method described by [43], and to visualize the membership of the individuals at each *K* with CLUMPAK [44]. According to the first method, an individual’s arithmetic mean (MedMeak and MaxMeak) or median (MedMeDK and MaxMedK) membership coefficient to a cluster should be greater than the threshold used. To assess the performance of the estimators, replicates of the Puechmaille method with a threshold 0.5 were run. The differentiation between the two populations needs to be larger than the threshold for them to be assigned in different clusters. Due to high computational demand of the software, a smaller dataset of 833 SNPs was used for STRUCTURE analysis (Supplement: Table S2). This dataset was obtained by repeating *populations* in stacks by keeping the loci that are present in all populations and at least 80% of individuals in each population.

Phylogenetic relationships among the populations were investigated using two different approaches. First, a maximum likelihood (ML) analysis in IQ-tree v2.2.0.8 [45] was performed after removing invariant sites in the alignment data that resulted in 13,581 SNPs. The algorithm *ModelFinder Plus* [46] built in IQ-tree was used to find the substitutional model. An ascertainment bias correction (+ASC) was implemented to the model testing. The best-fit model suggested by Bayesian Information Criterion was TVMe+ASC + R4 (Transversion model with equal frequency with FreeRate heterogeneity, and ASC). The model was then used to estimate the maximum likelihood topology with 98 randomly built parsimony trees as starting trees. Robustness of the phylogenetic hypothesis was assessed with 2000 replicates of ultrafast bootstraps (UFB) [47] and 2000 replicates of the SH-like approximate likelihood ratio test (SH-LRT) [48]. The recommendation that clades with UFB ≥ 95 and SH-LRT ≥ 80 can be considered as well supported was followed [47]. The most parsimonious tree suggested by the ML approach was visualized in FigTree v1.4.4 (http://tree.bio.ed.ac.uk/software/figtree/), and the tree was rooted at SPA population.

Isolation by distance (IBD) was estimated and evaluated by Mantel test (permutations = 999) on matrices of pairwise geographic distance (km) linearized genetic distance (F_st_/[1 − F_st_]) in package ade4 [49].

### Differentiation outlier scan

Since populations potentially could show adaptive genetic differentiation, we complemented our analysis of genetic structure with an analysis investigating genomic signatures of selection by performing an outlier approach. We did this by using pcadapt v.4.3.5 [37], which is a principal component-based analysis which identifies the SNPs most associated with the PC axes related to population structure. By using SNP z-scpres, pcadapt estimates the Mahalanobis distances between z-scores, and the first K principal components (PCs) related to population structure.

We analyzed the first 20 PCs graphically to retain the optimal K PCs (Fig. S3a). Based on the scree plot we retained 3 PCs (Fig. S3b, c) to calculate the test statistic and corrected for multiple testing using the Benjamini-Hochberg Procedure [50] with a false discovery rate (FDR) of 0.05.

We used BLAST to annotate the RAD-tags with SNPs putatively involved in local adaptation against the non-redundant protein database (blastx) restricting the search to insects only, retaining matches if they passed an e-value threshold of < 10^−5^ and at least ~ 70% query coverage (Supplement: Table S3).

We retrieved gene ontology terms for the identified outliers using g:Profiler [51], searching against *Drosophila melanogaster* GO terms, and using the native algorithm to correct for multiple testing employing a genome-wide threshold 0.05.

### Morphological analyses of wing shape

The right fore- and hindwing were cut off as close to the body as possible to obtain the whole wing. The wings were then placed in between two glass slides and photographed on graph paper as a length reference. The number of individuals used for the analysis are given in Table 1. After some individuals were discarded due to damage in the wing, we included 174 forewing and 196 hindwing samples for wing shape analyses.

**Fig. 2:**
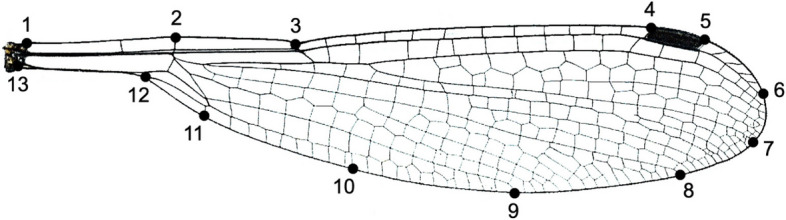
The 13 landmarks used to capture the shape on both the fore- and hindwings of the damselfly *Lestes sponsa*. Landmark 10 was a sliding (semi) landmark

Wing shape variation was analyzed with geometric morphometric techniques. Thirteen landmarks were placed along the outline of the wing, where major veins intersect the wing margin (Fig. 2). The landmarks were digitalized using the software tpsDig v.2.31 [52]. One of the coordinates (10) was a semi-landmark (Fig. 2). A Generalized Procrustes Analysis (GPA) was run separately for fore- and hindwings, thus removing the effects of position, rotation and isometric size by minimizing the total sums-of squared deviations of the landmark configurations from all specimens to the average landmark configuration [53]. In other words, landmark configurations were translated to the origin, scaled into unit centroid size, and rotated to minimize the total sums-of-squared deviations of the landmark coordinates from all specimens to the average configuration. The semi-landmark position was optimized by allowing it to slide along its tangent direction to minimize the Procrustes distance between the specimen’s landmark configuration and the average landmark configuration [54]. The landmark configurations (Supplement: Table S4 and S5 for forewings and hindwings, respectively) were also used to estimate wing centroid size, i.e., the squared root of the sum of squared distances between each landmark and the centroid of the configuration. Wing centroid size was log-transformed for further analyses and used as an estimate of body size. Previous studies in odonates showed a strong correlation between body size and wing size, including *L. sponsa* [16, 55, 56]. Unless otherwise stated, all geometric morphometric analyses were performed using the package geomorph v. 3.3.2 [57] for R.

Possible phylogenetic signal on wing shape variation was analyzed using the most parsimonious phylogenetic tree with the highest branch support. Wings were not available for all the tips of the phylogeny, so the tree was pruned for fore- and hindwings separately, resulting in 68 tips for forewings and 77 tips for hindwings. Phylogenetic signal was assessed on the Procrustes coordinates using the multivariate version of the K-statistic as proposed by Adams [58], by comparing the degree of phylogenetic signal in the dataset relative to what would be expected under Brownian motion. The significance of the multivariate K-statistic was obtained by 1000 random permutations of the shapes among the tips of the phylogeny. Phylogenetic signal was not significant for either fore- or hindwings (see Results), so subsequent analyses of wing shape did not consider the correlation with the phylogeny.

We performed a number of tests to inspect wing shape differences. First, the effects of population and wing centroid size were tested separately in fore- and hindwing shape. This was performed using Procrustes ANOVA on the Procrustes shape variables, with population entered as a factor and wing centroid size as a covariate. The interaction effect between population and wing centroid size was tested, and since it was not significant, the interaction effect was removed from the final models. The fitted models were also used to make pairwise comparisons of wing shape between populations. To obtain graphic representations of wing shape variation among the study populations, deformation grids were computed comparing each population mean wing shape to the wing shape of the entire dataset. The deformation grids were computed separately for the fore- and hindwing shape datasets and were enhanced × 10 times for ease of visualization.

Second, the latitudinal variation of wing size and shape was inspected. Latitude was log-transformed before entered in the models. The relationship between wing centroid size and latitude was studied using a linear model of wing centroid size on latitude, separately for fore- and hindwings. The linear models were compared to models including the quadratic effect of latitude. However, the quadratic models did not significantly explain more variation of wing centroid size (*P* > 0.05), so they were discarded. Procrustes ANOVA was used to determine the effects of latitude on wing shape variation, separately for fore- and hindwings. Models including only latitude were compared to models including also the quadratic term of latitude. The models including the quadratic term significantly explained more shape variation than the models including only the linear term (*P* < 0.05). Thus, the models including the quadratic term were retained. To graphically represent how overall wing shape varies along latitude, the regression scores proposed by Drake and Klingenberg [59] were used. Wing shape variations at the most southern and northern localities of our latitudinal gradient were visualized using again deformation grids, which compare any point in the morphospace to the average landmark configuration. The deformation grids were magnified × 20 times for ease of visualization.

Since a significant effect of wing centroid size was detected on wing shape, and wing centroid size varied along latitude (see Results), the latitudinal variation of the non-allometric component of wing shape was also explored. The residuals of the multivariate regression of wing shape on wing centroid size were used as the multivariate non-allometric component of wing shape. A Procrustes ANOVA was used to analyze the effects of latitude on the non-allometric component of wing shape for fore- and hindwings separately. Following a similar rationale as above, the model with the quadratic term of latitude was compared to the model with the linear term of latitude. Since the models including the quadratic explained significantly more shape variation (*P* < 0.05), the models with only the linear term were discarded. Further, to corroborate that wing shape differences due to latitude were not entirely driven by allometric effects, wing shape differences along the variation of wing centroid size were also estimated as deformation grids.

Finally, the regressions of wing shape variation on *F*_st_ values and geographic distance among populations were inspected. This analysis included all wing shape variation (allometric plus non-allometric component), because we were interested in observing the effects of *F*_st_ and proximity on overall wing shape variation. To obtain an estimate of dissimilarity of wing shape among populations, the Mahalanobis distances among all populations were computed using the function CVA in the Morpho package [60] for R. This was done separately for fore- and hindwings. The Mahalanobis distances obtained were then regressed separately on *F*_st_ values and geographic distance among populations. In addition, we performed pairwise comparisons of the Mahalanobis distances between populations. The *p*-values among the Mahalanobis distances were estimated using 1000 permutations of the pooled within-group covariance matrix. These p-values were estimated both uncorrected and corrected for multiple comparisons using the Holm’s correction [61].

## Results

### Genetic structure and phylogeny

Molecular diversity indices for each population based on 16,146 SNPs are presented in Table 1. *H*O ranged between 0.13 and 0.22, *H*E between 0.11 and 0.22, and *F*is between 0.049 and 0.100.

**Fig. 3:**
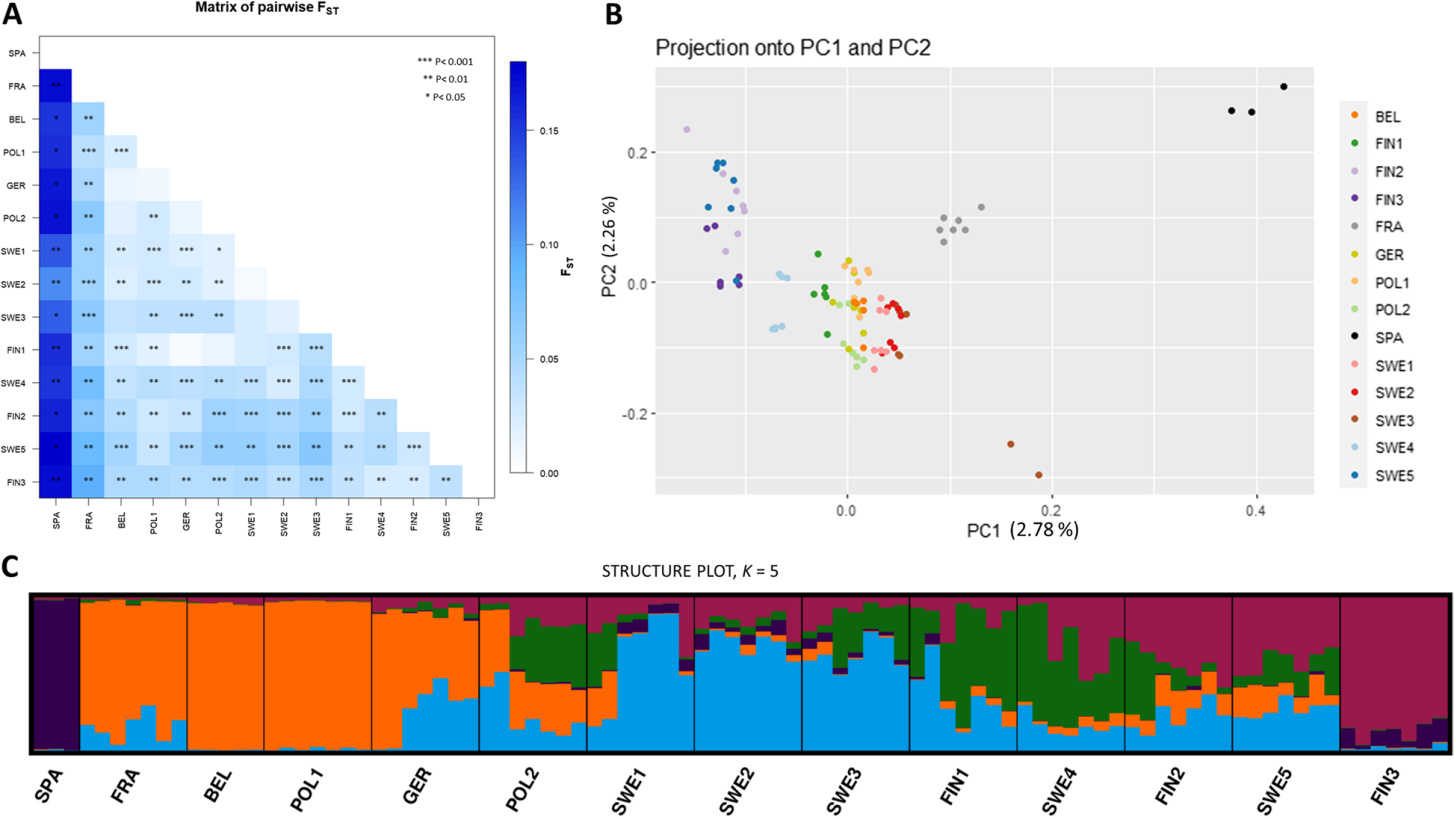
Results of the genetic structure analysis. **A** *F*_st_ comparison between 14 sampling locations. **B** PCA plots of 92 individuals. Samples were colored according to their sampling locations. **C** Genetic clusters according to STRUCTURE analysis at K = 5

Pairwise *F*_st_ values among the populations were low to moderate, ranging between 0.007 and 0.18, and the majority of the comparisons yielded a significant differentiation (81 out of 91 comparisons, *P* < 0.05, Fig. 3A). The highest differentiation was observed between the Spanish population (SPA) and all the other populations, while differentiation between the populations laying between the latitudes 50.83758 and 60.99038 (BEL, POL, GER: middle populations) and southern Scandinavia were the lowest.

The population subdivision analysis in STRUCTURE suggested that the most likely number of genetic clusters (*K*) were 3 or 6 according to Evanno Method (Fig. S4). According to the Puechmaille method [43] that uses four different statistics (MedMeak, MaxMeak, MedMeDK and MaxMedK), the *K* varied between 4 to 7 (Fig. S4). We accepted *K* = 5 as the most likely cluster (Fig. 3), since that structure plot was mostly supported by also other analyses, e.g., the PCA. Hence, the following more or less geographic clusters were suggested: (1) Spain (SPA), (2) some central European populations (FRA, BEL, POL1, and GER), (3) the southern Scandinavian populations (SWE1, SWE2 and SWE3), (4) northern Sweden and central Finland (SWE 4, SWE 5, FIN 1 and FIN 2), and finally (5) the northernmost Finnish population (FIN3). GER and POL2 were an admixture between the central European and the southern Scandinavian populations. Admixture was also observed between southern and northern Scandinavian populations (Fig. 3C, see Fig. S5 for clusters at other K values).

**Fig. 4:**
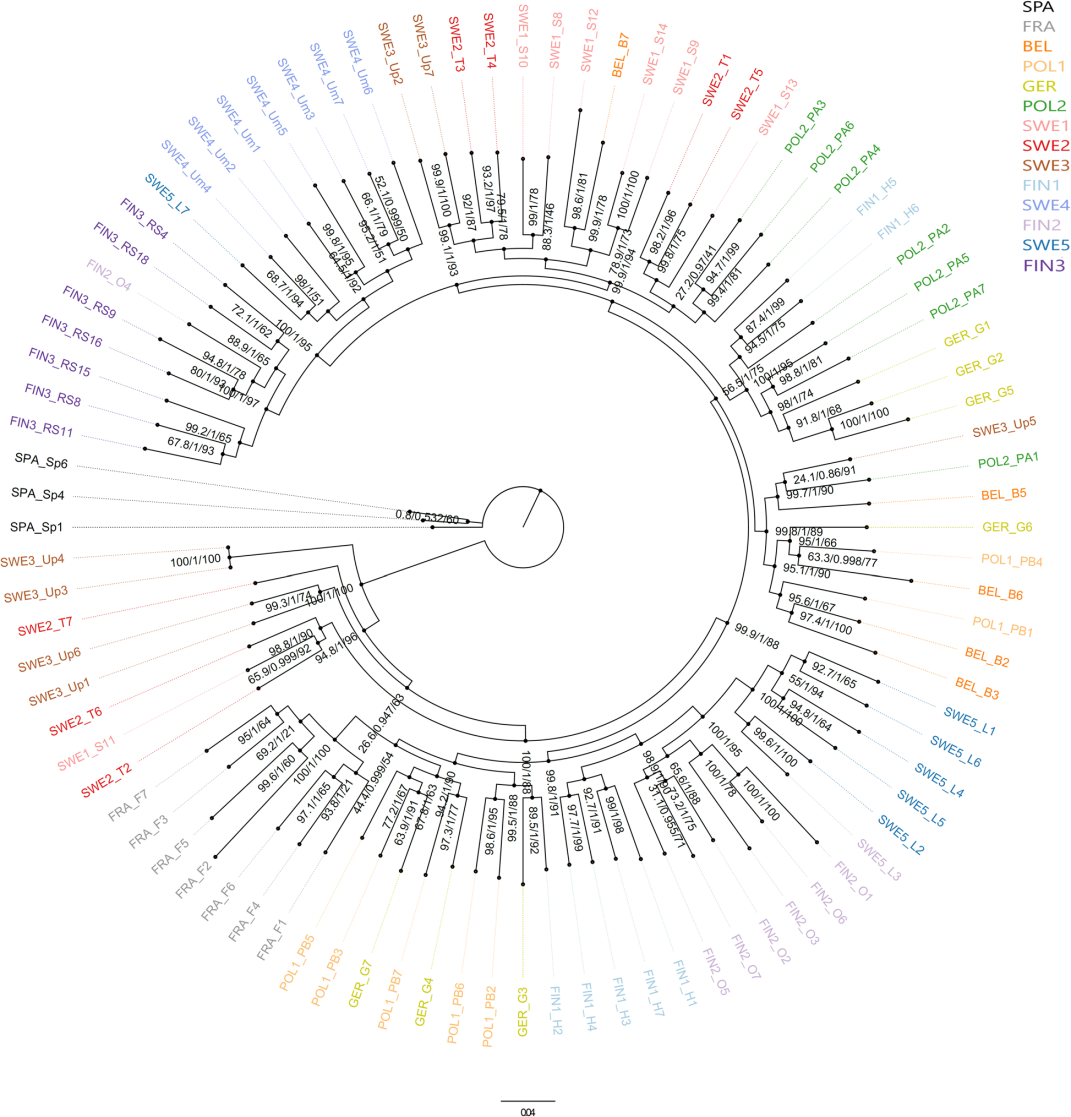
A maximum likelihood phylogenetic tree showing the evolutionary relationship between the individuals. Tips were colored according to their sampling locations. Node support values are UFB bootstrap, Bayesian posterior probability, and SH-LRT bootstrap values, respectively

The PCA plot supported the structure patterns suggested by both *F*_st_ and STRUCTURE analysis. The SPA population was the most distinct population, and FRA population also formed a distinct cluster (Fig. 3B). The central European and southern Scandinavian populations were almost indistinguishable from each other (Fig. 3B). In addition, the northern Finnish populations (FIN 2and FIN 3) and the northernmost Swedish population (SWE 5) formed a cluster. FineRadStructure did not find a strong genetic differentiation among the populations, yet supported the results of the other analysis (Fig. S6). The result of the phylogenetic analysis mirrored that of the cluster and PCA analyses, and the majority of the branches had high support (UFB ≥ 95 and SH ≥ 80) (Fig. 4). The tree showed that the Spanish (rooted in analysis), French and south Swedish populations were the ancestral ones with northern Swedish and Finnish populations being the most derived ones. The tree also suggested that population origin and phylogeny did not show a perfect match. For example, some northern Swedish individuals are in the clade with Central European populations and a Belgian individual is at the Swedish clade, suggesting dispersal occurring between the populations.

**Fig. 5:**
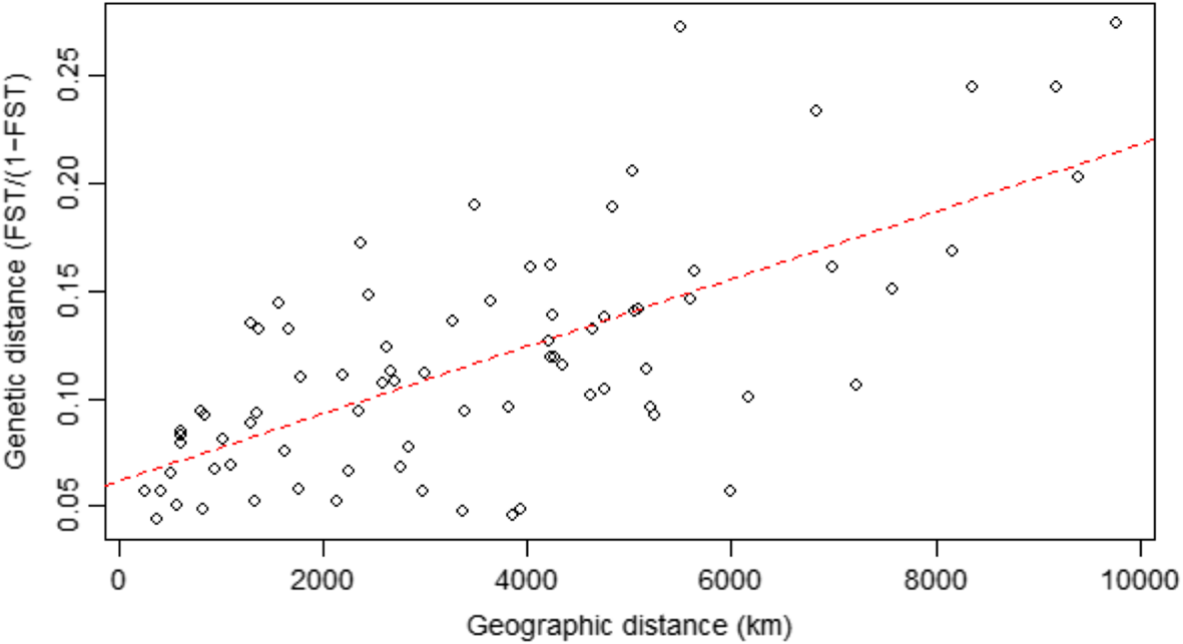
Correlation between pairwise geographic distance and linearized fixation index (*F*_st_) among the 14 study populations

Mantel test between linearized *F*_st_ and geographic distance showed a strong correlation between genetic and geographical distance with high significance (*R* = 0.68, *P* = 0.001) supporting a pattern of IBD (Fig. 5).

### Differentiation outlier scan

Pcadapt identified 1598 SNPs passing the FDR cutoff (Fig. S7a), with the test statistic and *p*-values following the expected chi-square and uniform distribution (Fig. S7b-d), putatively involved in local adaptation. Of the 1598 RAD-tags, 233 matched a unique insect protein, with 102 of the hits matching uncharacterized, unknown or hypothetical proteins. Significantly enriched GO terms had biological processes involved in response to stimulus, response to stress and cell morphogenesis (Table S6).

### Wing morphology

There was not a significant phylogenetic signal for either forewings (*K* = 0.498, *P* = 0.312) or hindwings (*K* = 0.088, *P* = 0.686). Thus, subsequent analysis did not consider phylogenetic relationships among individuals.

When we used the Procrustes distances, wing shape differed significantly among populations and there was a significant allometric effect of wing centroid size (forewings: population *F*_13, 173_ = 2.947, *P* < 0.001; wing centroid size *F*_1,173_ = 2.768, *P* = 0.027; hindwings: *F*_13, 195_ = 4.130, *P* < 0.001; wing centroid size *F*_1,195_ = 4.339, *P* = 0.005; Fig. 1, Fig. S8). However, corrected pairwise comparisons showed no significant differences among pairs of populations (Table S7).

**Fig. 6:**
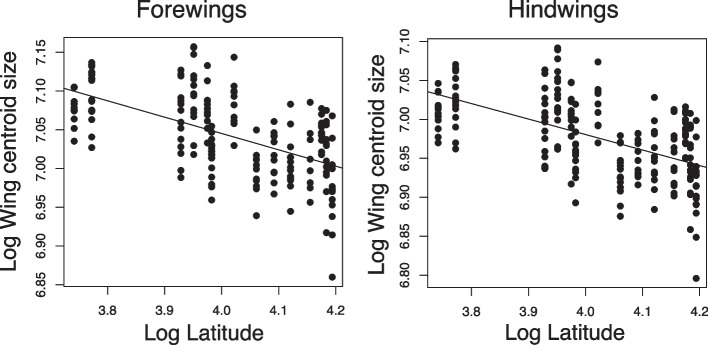
Variation of log wing centroid size over log latitude for fore- (left) and hindwings (right). The regression line shows a linear fit (forewings adjusted *R*^*2*^ = 0.317, *P* < 0.001; hindwings adjusted *R*^*2*^ = 0.319, *P* < 0.001)

Wing centroid size significantly decreased with latitude, both for fore- (adjusted *R*^*2*^ = 0.317, *P* < 0.001) and hindwings (adjusted *R*^*2*^ = 0.319, *P* < 0.001). Individuals thus showed smaller sizes towards more northern populations (Fig. 6).

**Fig. 7:**
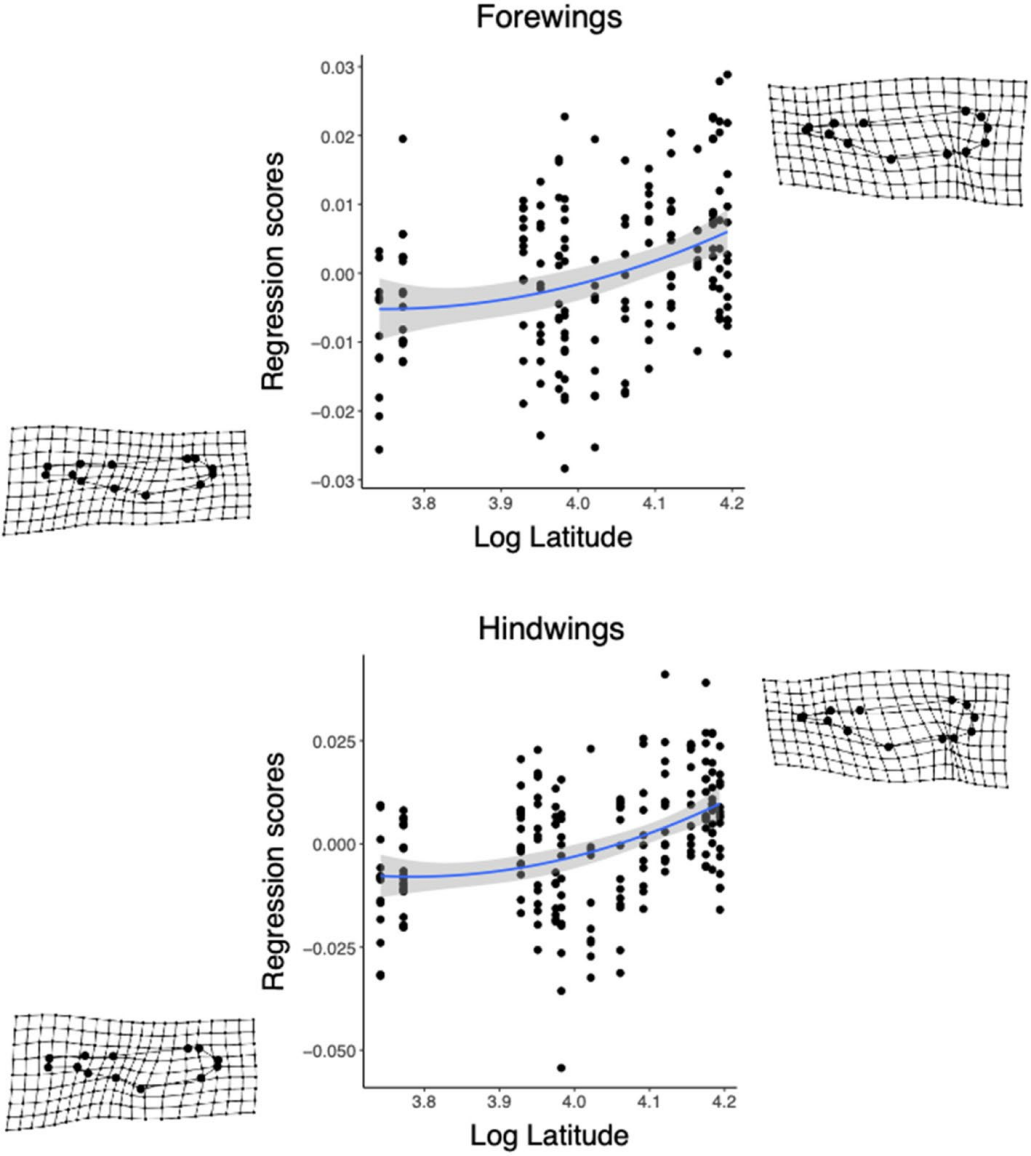
Variation of wing shape (shown as regression scores) over log latitude for fore- (top) and hindwings (bottom). The regression line shows a quadratic fit, and the shaded region represents the 95% CI around the line. The deformation grids show the variation of wing shape at the most top right and most bottom left individuals on the plot. Deformation grids are magnified ×20 times

Forewing shape variation was significantly explained by latitude and its quadratic term (latitude: *R*^*2*^ = 0.032, *F*_1,173_ = 5.749, *Z* = 2.952, *P* = 0.001; latitude^2^: *R*^*2*^ = 0.032, *F*_1,173_ = 5.807, *Z* = 2.971, *P* = 0.001), and the same was true for hindwing shape (latitude: *R*^*2*^ = 0.032, *F*_1,195_ = 6.853, *Z* = 2.966, *P* = 0.002; latitude^2^: *R*^*2*^ = 0.033, *F*_1,195_ = 7.036, *Z* = 2.990, *P* = 0.002) (Fig. 7). Both for fore- and hindwings, the extreme of wing shape variation at the northernmost population showed wider wings that were more compressed on the apical half along its length (Fig. 7). On the contrary, at the southernmost population, the extreme of wing shape variation showed more slender wings with a more compressed first half along the length (Fig. 7).

The allometric shape variation with wing centroid size was significant (Fig. S9), but subtle compared to wing shape variation along latitude (Fig. 7). Despite the effects of size on wing shape, the non-allometric component of wing shape (residuals of regression scores from the allometric line) still showed a significant quadratic (although weak) relationship with log latitude in both fore- (latitude: R^2^ = 0.032, F_1,173_ = 5.645, Z = 2.913, *P* = 0.001; latitude2: R^2^ = 0.032, F_1,17_3 = 5.681, Z = 2.924, P = 0.001) and hindwings (latitude: R^2^ = 0.031, F_1,195_ = 6.316, Z = 2.810, *P* = 0.003; latitude2: R^2^ = 0.031, F_1,195_ = 6.423, Z = 2.827, P = 0.003).

Pairwise Mahalanobis distances showed significant differences in wing shape among populations when inspecting the uncorrected *P*-values, but not when inspecting the Holm’s corrected P-values (Table S8). The Mahalanobis distances were correlated to *F*_st_ and geographic distances among populations, and linear regressions showed that wing shapes were more different between populations with longer the geographic distance between them (forewings: adjusted *R*^2^ = 0.308, *P* < 0.001; hindwings: adjusted *R*^2^ = 0.392, *P* < 0.001), and with higher *F*_st_ values (forewings: adjusted *R*^2^ = 0.349, *P* < 0.001; hindwings: adjusted *R*^2^ = 0.301, *P* < 0.001).

## Discussion

We predicted that wing shape should not show a phylogenetic signal, because we expect local adaption in wing shape across the populations. We found that wing shape differed among *L. sponsa* populations in Europe and that no phylogenetic signal was present in wing shape variation. Our estimates of heterozygosity and inbreeding coefficient suggested that none of the populations were inbred and thus genetic drift is unlikely to have caused the observed differences in wing shape among populations. The majority of the *F*_st_ values between populations were significant and the *F*_st_ showed an isolation by distance relationship. In addition, our outlier analysis on SNPs suggested that some regions of the genome (1598 out of 16,146: about 10%) are putatively shaped by selection. This suggests that neutral processes such as genetic drift are not driving 100% of the observed genetic differentiation. Our GO enrichment analysis showed that many of these are involved in stress and physiological processes. We speculate that many of these are associated with the climatic and time stress conditions differences experienced by the different populations. However, we note that no specific wing morphology SNPs were found.

Taken together, these findings suggest that local selection pressures differ among populations. Wing shape variation was also found to increase with geographic distance among populations, and with *F*_st_ values. Moreover, our results also showed that wing shape variation correlated very weakly with latitude (explaining just about 3% of the total wing shape variation, even when accounting for allometric effects), suggesting that local selection pressures do not seem to follow a latitudinal gradient. These results agree with a previous study in the same species, albeit using another dataset [16]. However, we note that other aspects of wing morphology, like wing size, showed a strong correlation with latitude (explaining about 32% of wing size variation), mirroring earlier findings of a negative relationship of structural body size and latitude in *L. sponsa* [62], but note that Outomuro et al. [16] found a U-shaped pattern.

We estimated phenotypic wing shape of wild caught individuals and hence the observed wing shape variation was probably affected by a combination of genetic and environmental effects. In our study species, photoperiod imposes time constraints impacting larval growth, developmental time, and adult morphology [21, 23, 63, 64]. In a recent study, Johansson et al. [23] showed that wing shape differences between northern and southern populations of *L. sponsa* were composed of genetic and plastic responses to photoperiod and temperature in the larval rearing environment. However, that study [23] was only investigating two populations (north and central), which makes it difficult to compare the observed latitudinal patterns of wing shape to the present study. Nevertheless, the two major abiotic variables that differ along the latitudinal range and that potentially could affect wing shape in this study are photoperiod and temperature. Our results suggest that these variables do not have a strong impact on phenotypic wing shape, because latitude only affected a small amount of the variation in wing shape (see also [16] for a similar result). Instead, other unexplored variables seem to be more important.

The absence of a clear latitudinal wing shape pattern in our study differs from that found in many other insects, where a latitudinal cline in wing morphology has been found [15, 65]. In addition, in insects, temperature per se has been shown to impact wing shape via optimization of flight under different thermic conditions [66]. For example, laboratory studies in Diptera have shown that a large part of the wing shape variation observed along latitudinal gradients is shaped by temperature [17, 18]. However, we note that some studies in dipterans do not follow that pattern [14]. We also note that our wing shape analysis did not distinguish between genetic and environmental variation including plasticity. A recent meta-analysis showed a moderate relationship between genomic differentiation and phenotypic differentiation [67], and in another study we found an alignment between phenotypic plasticity and genetic variation [23]. These two studies suggest that some of the observed wing shape variation may have been caused by phenotypic plasticity that might be adaptive.

Besides photoperiod and temperature, dispersal is another important factor that can impact wing shape variation along latitudinal gradients. Since many insects including odonates [68] have expanded their range in the last decades, we might predict dispersal phenotypes at northern expanding populations [25, 69–71]. We expected a slender wing shape in northern populations at range margins because a slender wing might be beneficial for dispersal in damselflies [25], but we did not find support for this. Indeed, the wing shape pattern with regard to latitude was in the opposite direction with slender wings in the south and wider wings in the north, although we note the regression only explained 3% of the variation. In addition, visual inspection of wing shape showed large variation among populations in the north as well as in the south. One explanation for the lack of support for our hypothesis is that the adaptive benefit of an optimal wing shape for dispersal might be selected against very effectively once a population is established at a latitude [72, 73]. Since *L. sponsa* has a one-year life cycle in Europe [26], adaptation to local selection on wing shape could work fairly fast across the studied populations.

Certain local selection pressures might also contribute to the observed wing shape variation among populations. Previous studies showed that sexual selection and predation risk can impact wing shape in *L. sponsa* [19], and other species of damselflies [13, 74]. There is certain evidence that sexual selection is stronger at higher latitudes [75–77], and that predation is lower at higher latitudes [78, 79]. Our results did not show variation in wing shape following a clear latitudinal pattern that would support such an expectation. However, local sexual selection and predation pressures could still impact the observed differences in wing shape among populations, just not following a latitudinal cline. In summary, we suggest that the variation in wing shape among population is probably a result of spatial and or between year variation in local selection pressures such as e.g., sexual selection and predation, different from factors correlated with latitude such as time constraints or temperature. Some fraction of this variation could also be due to environmental effects, e.g., variation in temperature during the larval stage which has been shown to affect insect wing morphology [66].

In contrast to wing shape, wing centroid size which is correlated with body size in *L. sponsa* [16, 55, 56], showed a strong correlation with latitude suggesting that phenotypic body size is much smaller at higher latitudes. Studies on body size gradient of insects show a mixture of positive and negative size relationship with latitude [80]. One reason for this mix of results is probably a confounding effect of voltinism. Considering univoltine species such as *L. sponsa*, the general pattern is a decrease in size with latitude [16, 62], which we also found here. The main reason might be time constraints, that is, a shorter growth season at higher latitudes, which results in less time available for growth and development and thus emergence at a smaller size [63]. In contrast, semivoltine species are less affected by seasonal time constraints due to a prolonged development time, and might therefore show different patterns in size with regard to latitude.

Our analyses suggested a significant, but relatively weak population structure and a moderate degree of genetic variation within the area investigated. The observed weak population structure matches findings in other well dispersed damselfly species across Europe [81, 82]. Our structure analysis suggested five clusters of populations. We rooted our phylogeny in Spain, since many studies have suggested that a refugia during the last ice age occurred in southern Europe. Within the clusters identified, the Spanish and the northernmost Finnish populations came out as two clear clusters. The Spanish population seemed to be very isolated as was also suggested by the PCA analysis and the *F*_st_values. This is not surprising since the Pyrenees might work as a strong dispersal barrier. Studies on other species, including odonates, have shown a similar isolation pattern of Spanish populations [81–83]. The northernmost Finnish population was also distinct from the other populations, including the close north Scandinavian populations. One reason for this distinct cluster could be that this population might be a result from an eastern migration route since the last ice age [83]. An eastern invasion route of north Scandinavian species has been found in other species such as frogs [84] and shrews [85]. However, sampling regime covering a wider geographical range towards the east including the Balkans is needed to confirm such hypothesis. Finally, our phylogeny and PCA analyses supported the structure with French, German and Polish populations being most closely related to the Spanish ones. Thus, we found some support for our prediction that the phylogeny of the sampled populations should reflect a dispersal from the glacial refuges: the ancestral population stems from the southern latitudes, whereas the northern populations derive from it. However, some Swedish individuals were closely related to the Spanish population and in addition some Polish, Belgian and German individuals were situated in between Swedish and Finnish individuals in the phylogeny. This suggests again significant gene flow within the large study area.

In summary, we found phenotypic differences in wing shape amongst *L. sponsa* populations across Europe. These differences might be a result of local selection pressure since *F*_st_ differed among the majority of the populations, and since our outlier analyses on SNPs identified signature of selection. However, more analyses such as a *Q*_st_/F_*st*_ comparison are needed to confirm this suggestion. This phenotypic difference in wing shape difference was not an effect of phylogeny, and it is also probably not driven by latitudinal effects such as photoperiod or temperature. The absence of a clear latitudinal gradient in wing shape adds to many recent studies that also have not found a clear relationship between wing shape and latitude in insects [86].

### Supplementary Information

**Additional file 1.**

**Additional file 2: Table S1.** Description of the data and file structure: This VCF file includes the final dataset (16,146 SNPs) for 92 individuals from 14 populations. The content of each column is described in the beginning of the file.

**Additional file 3:** **Table S2.** Description of the data and file structure: This VCF file includes the final dataset (833 SNPs) for 92 individuals from 14 populations. The content of each column is described in the beginning of the file.

**Additional file 4:** **Table S3.** Results from BLAST to annotate the RAD-tags with SNPs putatively involved in local adaptation against the non-redundant protein database (blastx) restricting the search to insects only, retaining matches if they passed an e-value threshold of < 10^-5^ and at least ~70% query coverage.

**Additional file 5:** **Table S4.** Description of the data and file structure: This file in TPS format contains data for 174 forewing samples, and each 13 landmarks for each forewing. "ID" represents the sample name. Only a subset of the samples were used in the genetic analyses. Those names can be tracked using the additional file 1 or 2.wing.

**Additional file 6:** **Table S5.** Description of the data and file structure: The TPS file contains 196 hindwing samples, and 13 landmarks for each hindwing. "ID" represents the sample name. Only a subset of the samples were used in the genetic analyses. Those names can be tracked using the SNP (additional file 1 and 2). If needed, we can provide the researchers with the picture of each wing.

**Additional file 7:** **Table S6.** List of enriched GO terms that had biological processes involved in response to stimulus, response to stress and cell morphogenesis.

**Additional file 8.**

**Additional file 9.**

## Data Availability

Demultiplexed raw DNA sequences are archived in the NCBI Sequence Read Archive (BioProject ID: PRJNA971006), link: https://www.ncbi.nlm.nih.gov/bioproject/PRJNA971006. Wing morphology is provided in Supplementary Table S4 and S5.
