# Supplementary material for: Phylogeography and phenotypic wing shape variation in a damselfly across populations in Europe
Source: BMC Ecol Evol. 2024 Feb 3;24:19. doi: 10.1186/s12862-024-02207-4 (PMC10838002; doi:10.1186/s12862-024-02207-4)
Supplement: Supplementary file 1 — Additional file 1. [file 12862_2024_2207_MOESM1_ESM.docx]

**Supplementary Figures**

**Phylogeography and phenotypic wing shape variation in a damselfly across populations in Europe**

**Y. Yildirim^1^, D. Kristensson^1^, D. Outomuro^2^, D. Mikolajewski^3^, Rödin Mörch, P., S. Sniegula^4^, F. Johansson*^1^**


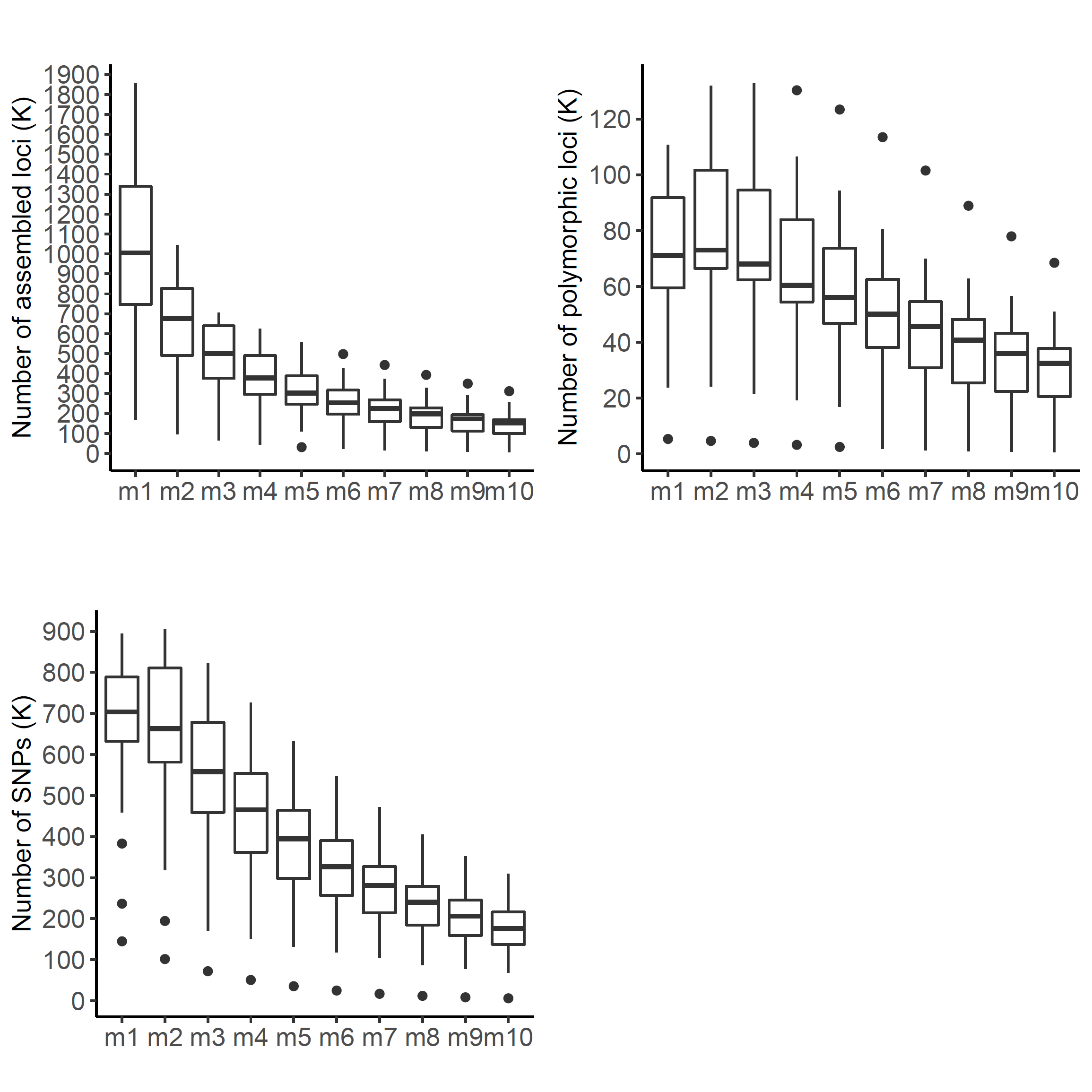


Figure S1. Results obtained from the parameter optimization. Plots show the number of assembled loci, polymorphic loci and SNPs for different parameter settings for m values (minimum number of raw reads required to form a stack/putative allele) ranging from 1 to 10. To select parameter setting for m, the denovo pipeline was run while fixing M = 2 (M = number of mismatches allowed between stacks/putative alleles to merge them into a putative locus) and n = 1 (n = number of mismatches allowed between stacks/putative loci during construction of the catalog). The stabilization of the plots occurred at m = 6. Therefore, we choose this value for the whole dataset.


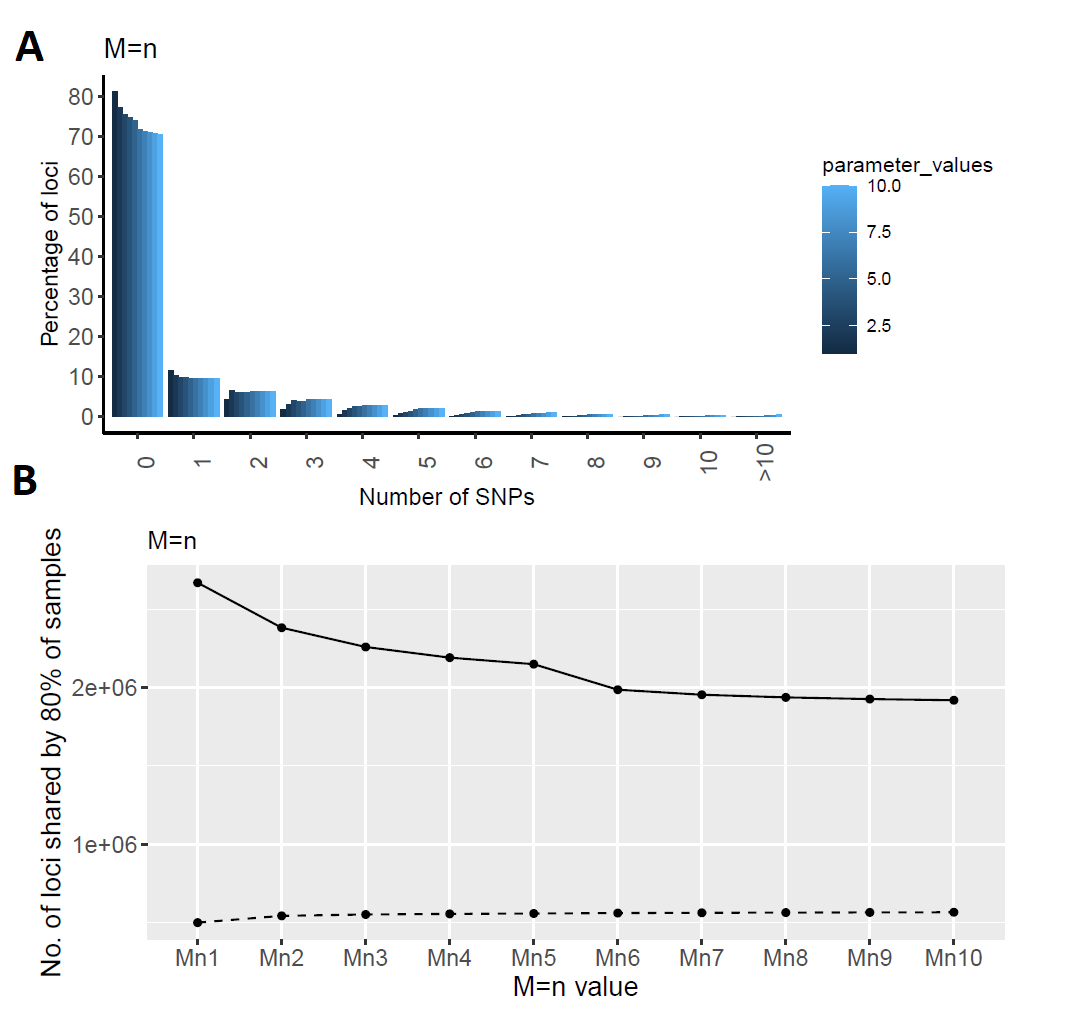


Figure S2. Results obtained from the parameter optimization. (A) The plot shows the distribution of number of SNPs per locus and (B) number of loci shared by 80% of samples for iterated values of M and n following the instructions in (Rochette & Catchen, 2017). The stabilization of the plots occurred at M = n = 6. To select parameter setting for M and n, the de novo pipeline was run while fixing m = 3 and keeping M = n for a range of 1 to 10.


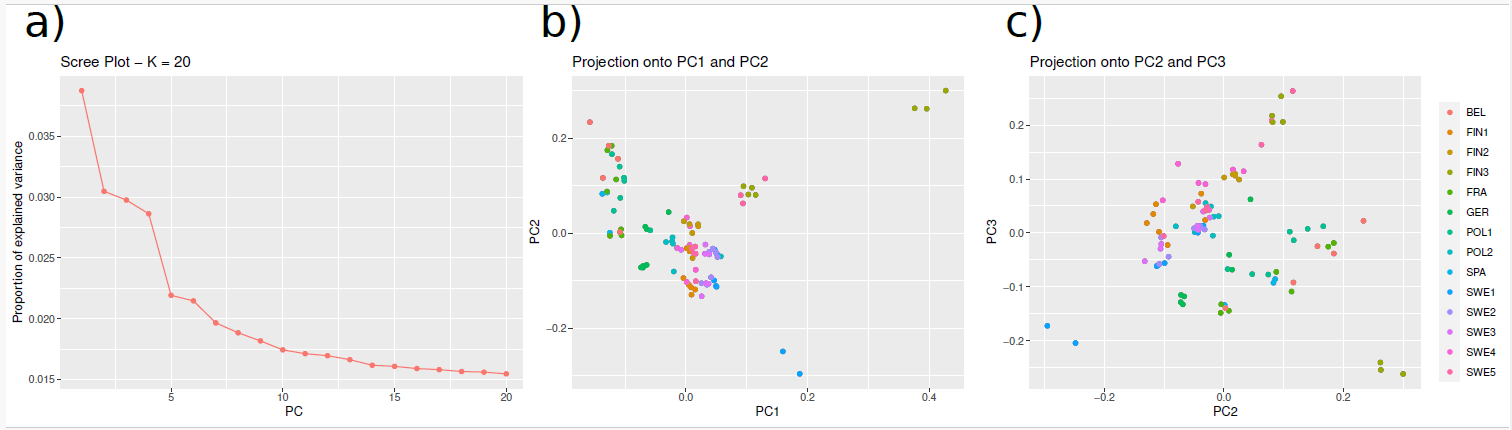


Fig. S3a) Scree-plot showing the decrease of explained variance with increasing principal components. Cutoff for analysis set at the elbow at K3. b) PCA score plot PC1 and PC2, c) PC2 and PC3.


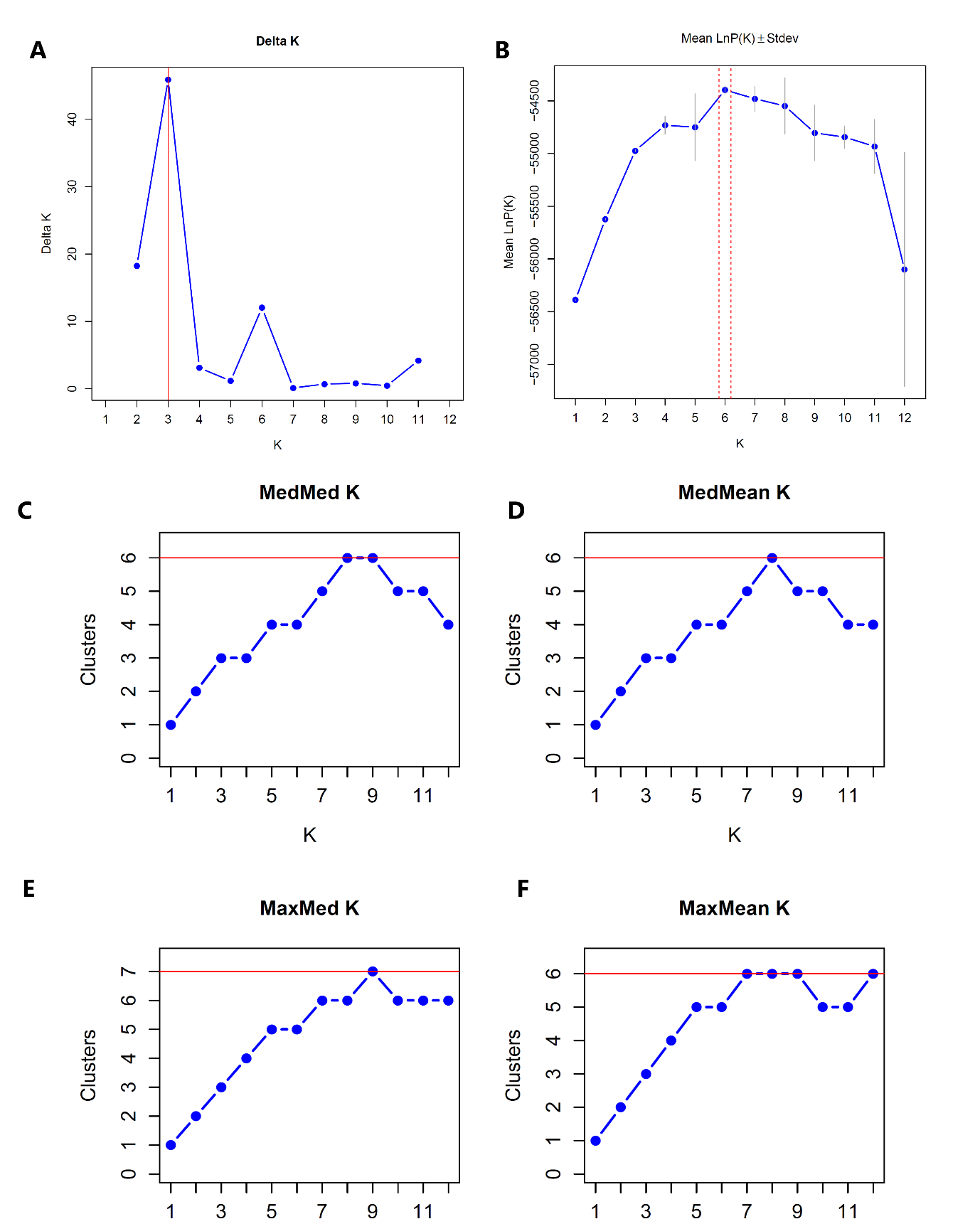


Figure S4. Analyses of STRUCTURE outputs following Evanno test and Puechmaille method (Puechmaille 2016) for 833 SNP markers. Evanno methods: A) ΔK and B) Mean LnP(K). Puechmaille methods: C) MedMedK, D) MedMeaK, E) MaxMedK and F) MaxMeaK.


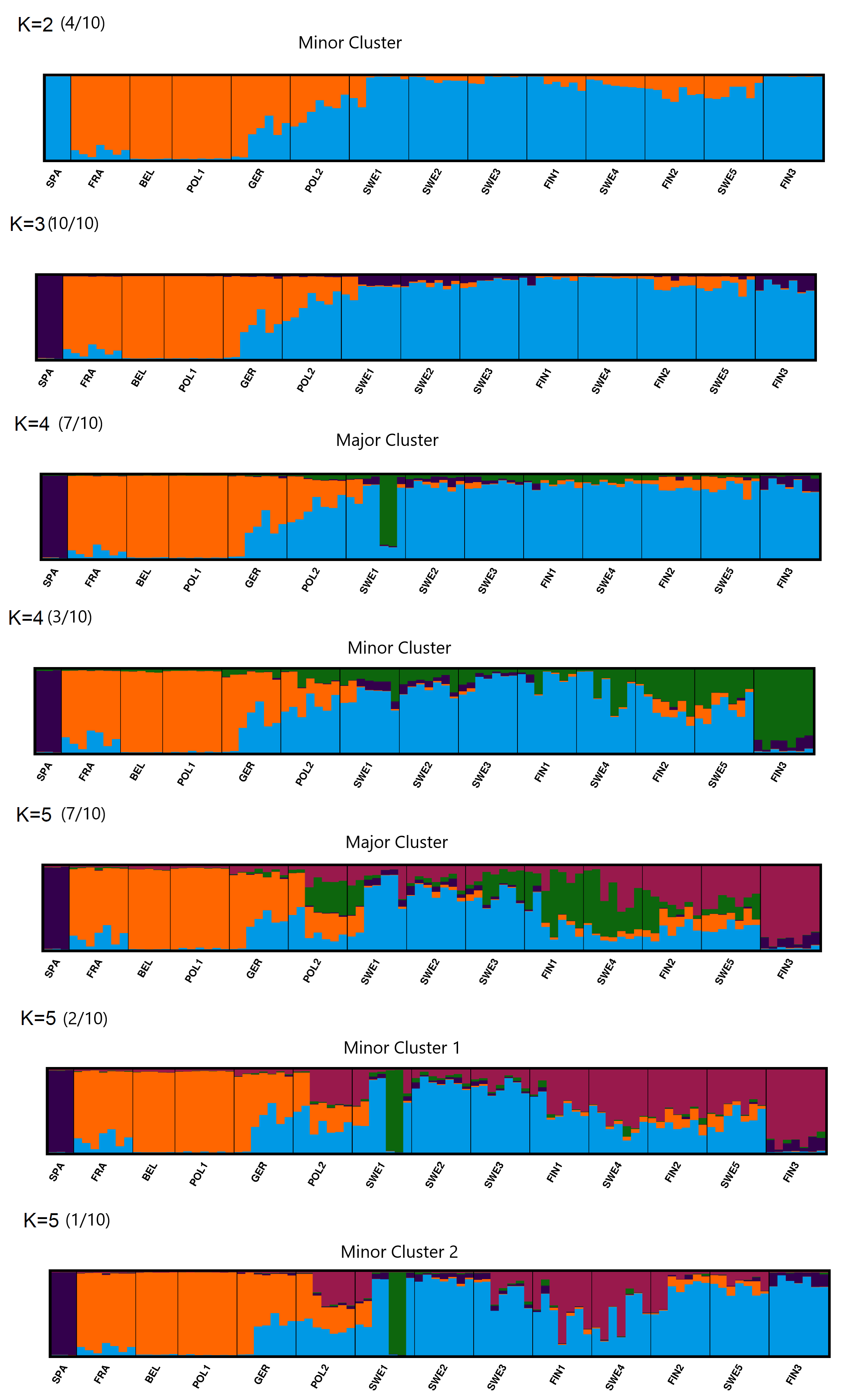


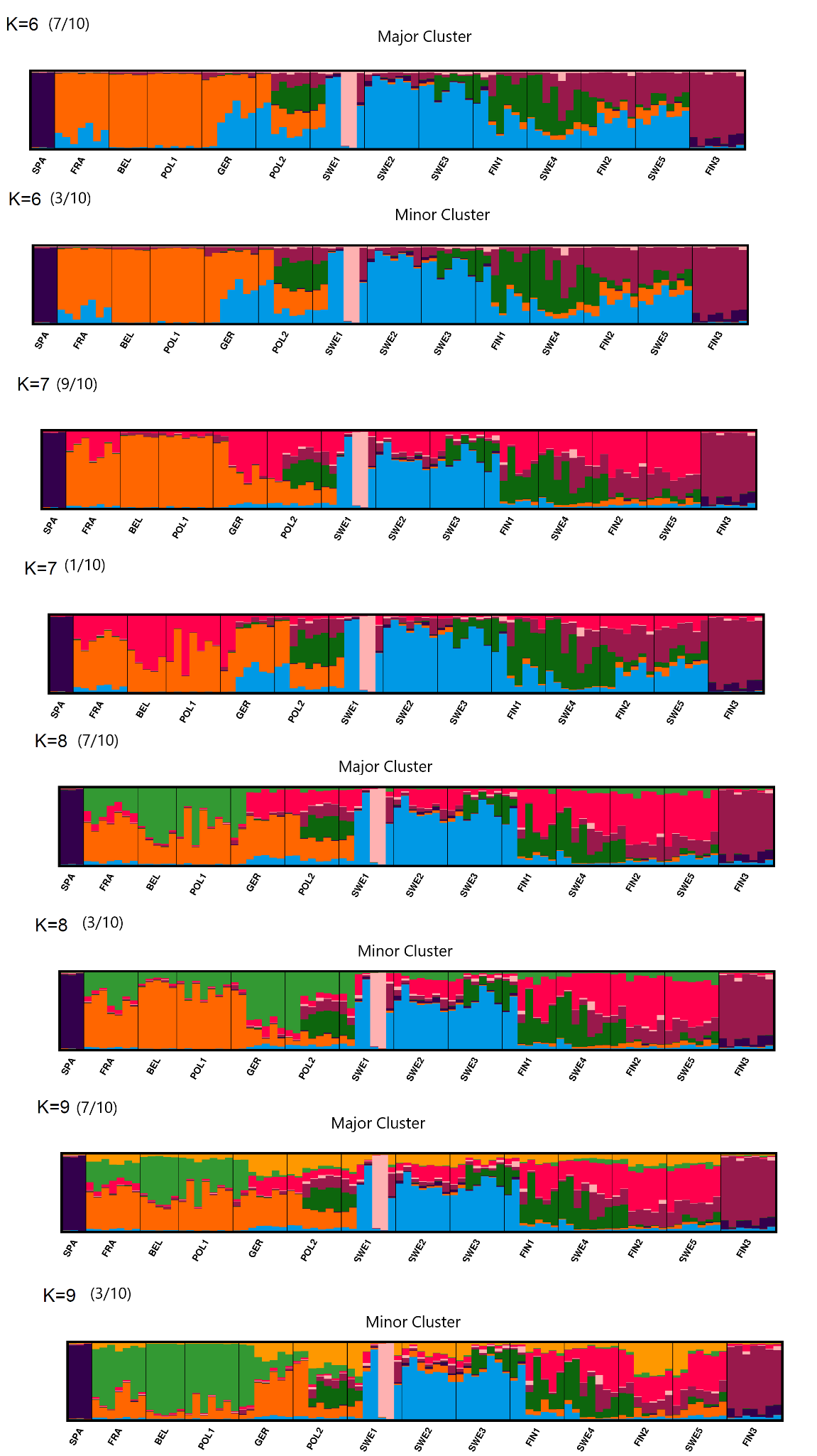
Figure S4. (continued)


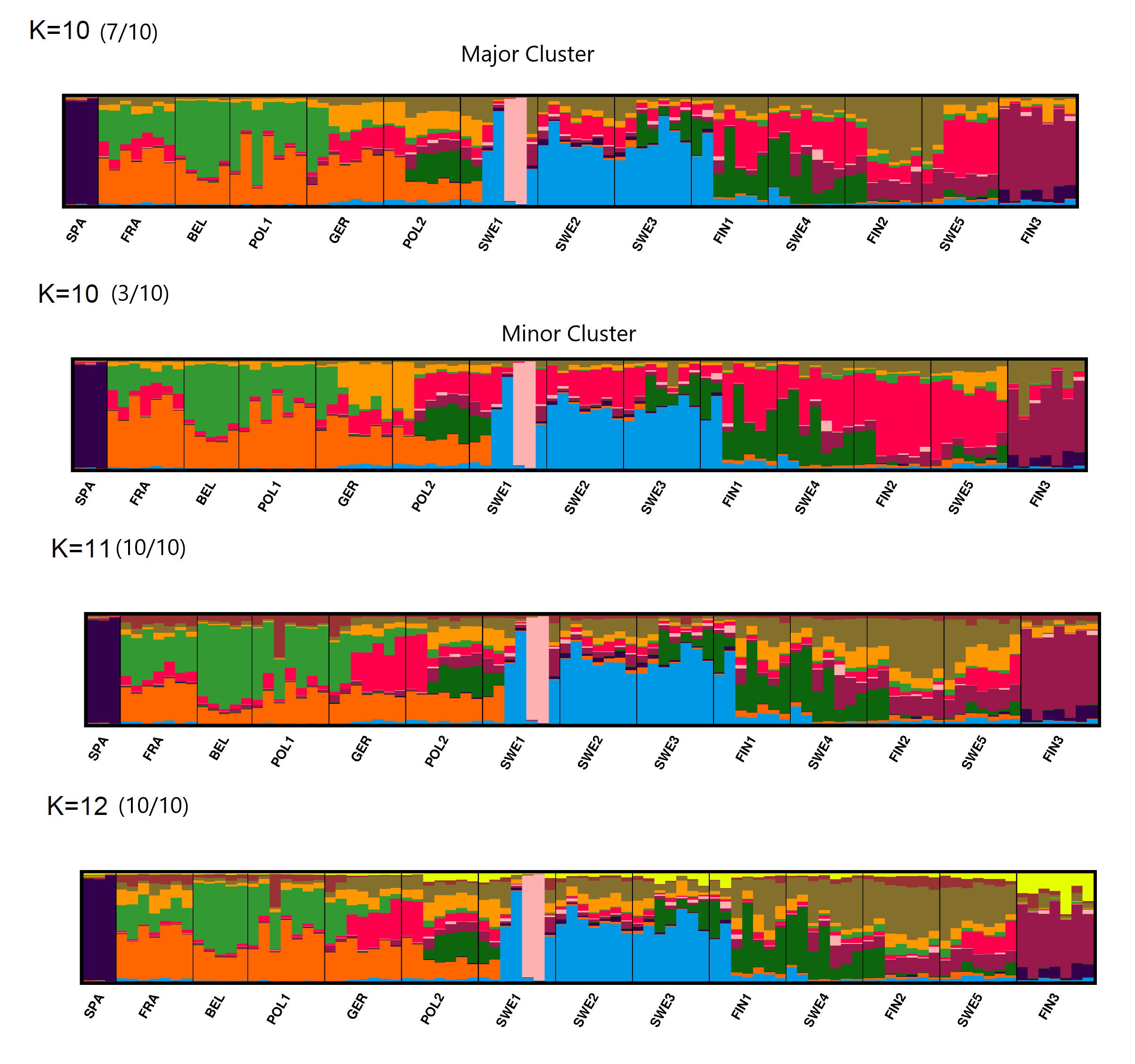


Figure S5. Genetic clusters according to STRUCTURE analysis at each tested K (K=2-12). Clusters supported by both the majority and the minority of the analyses (10 repetitions) are presented.


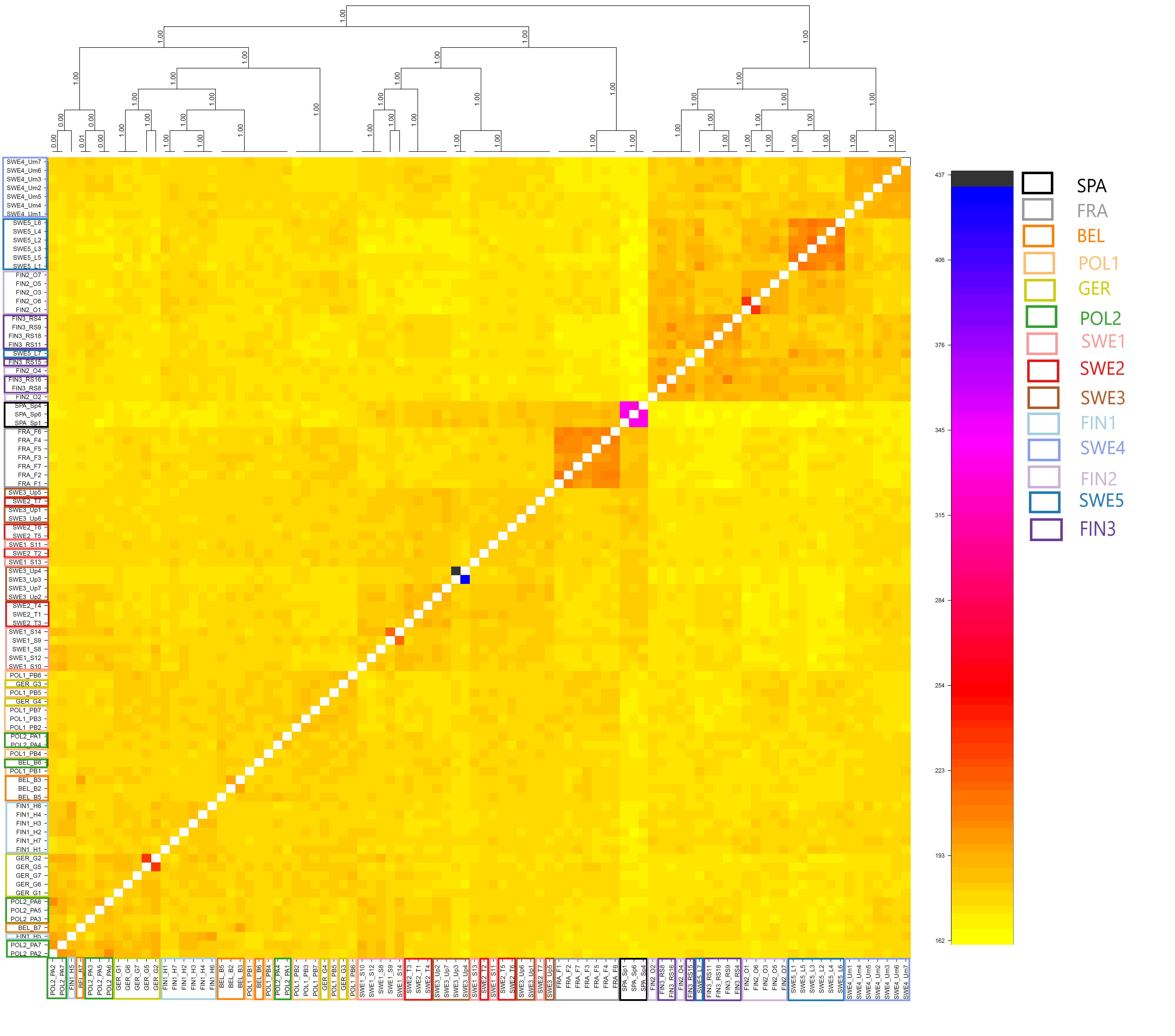


Figure S6. Co-ancestry matrices according to fineRadStructure analyses based on genetic similarity. Darker colors mean higher levels of co-ancestry, and less divergence between two individuals. Frame colors show the same sampling locations.


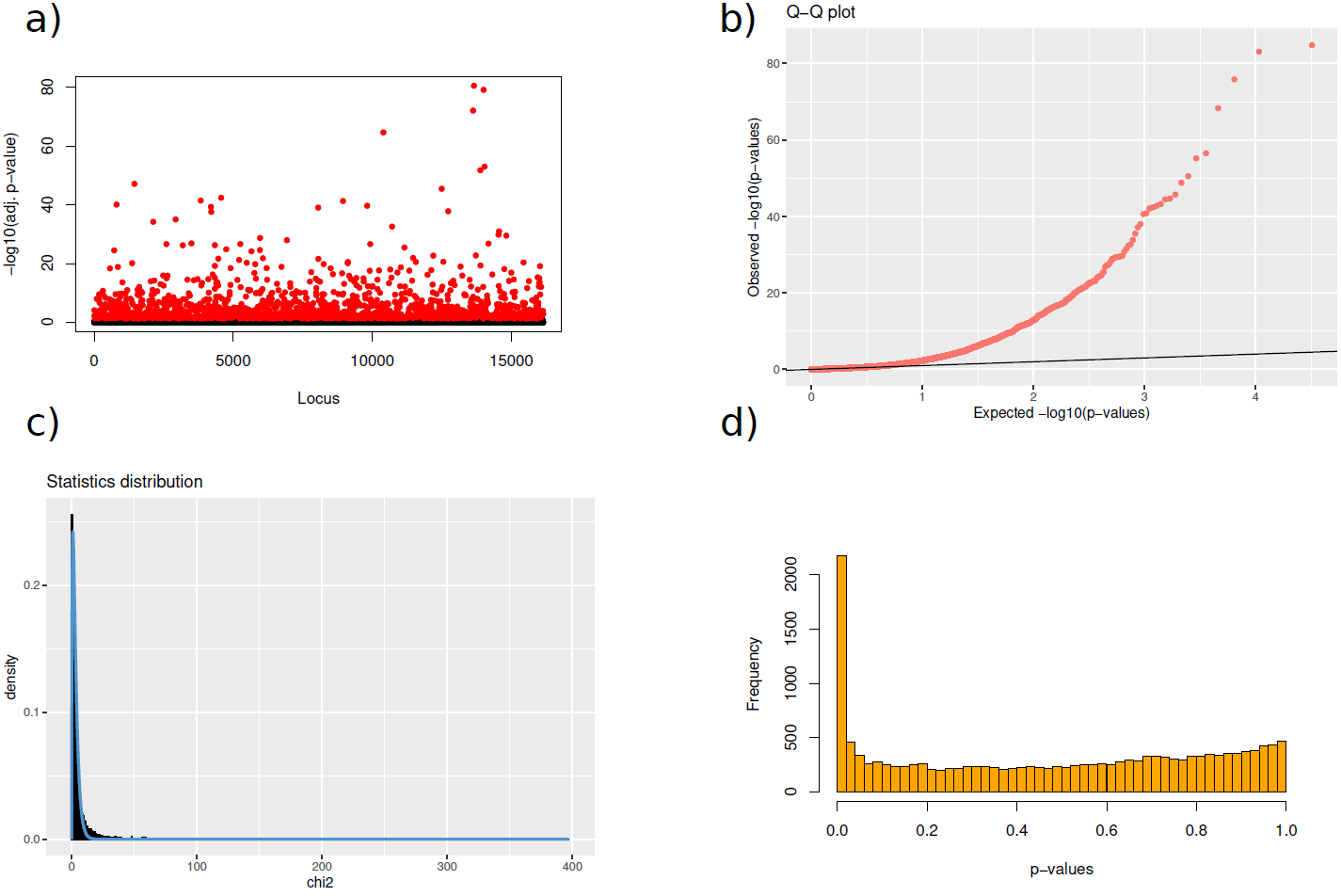


Fig. S7 a) Manhattan plot showing the adjusted p-value for SNPs passing a FDR of 0.05 in red. b) QQ plot showing the deviation of observed versus expected p-values for the outliers. c) A mostly uniform distribution of p-values including an excess of small pvalues (outliers). d) A distribution of the test statistic showing high Chi-square values in the tail corresponding to the outliers.


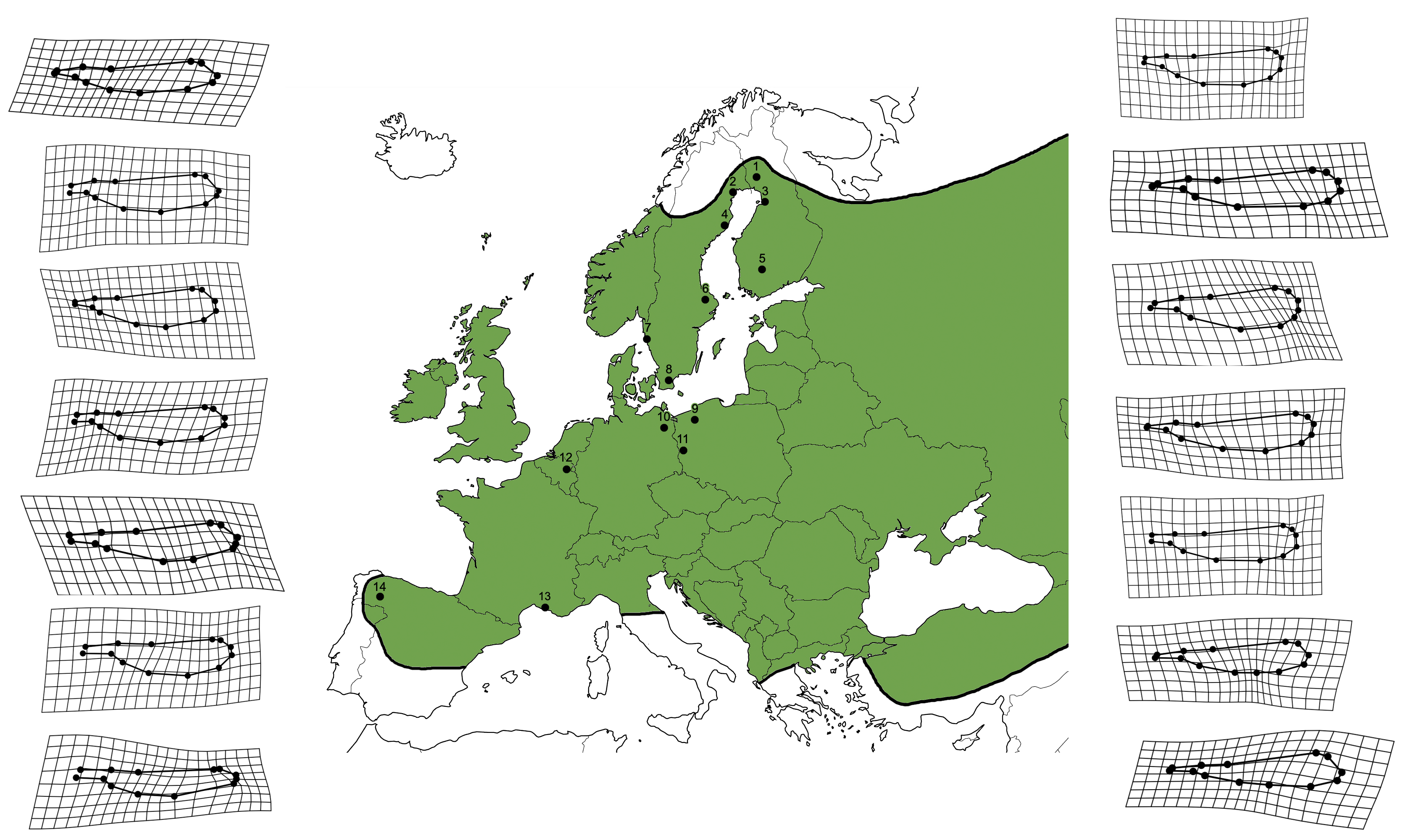


Figure S8. Deformation grids of hind wing shape variation among the 14 populations sampled. Grids are enhanced x10 times for ease of visualization. Information for creating he map was taken from [20].


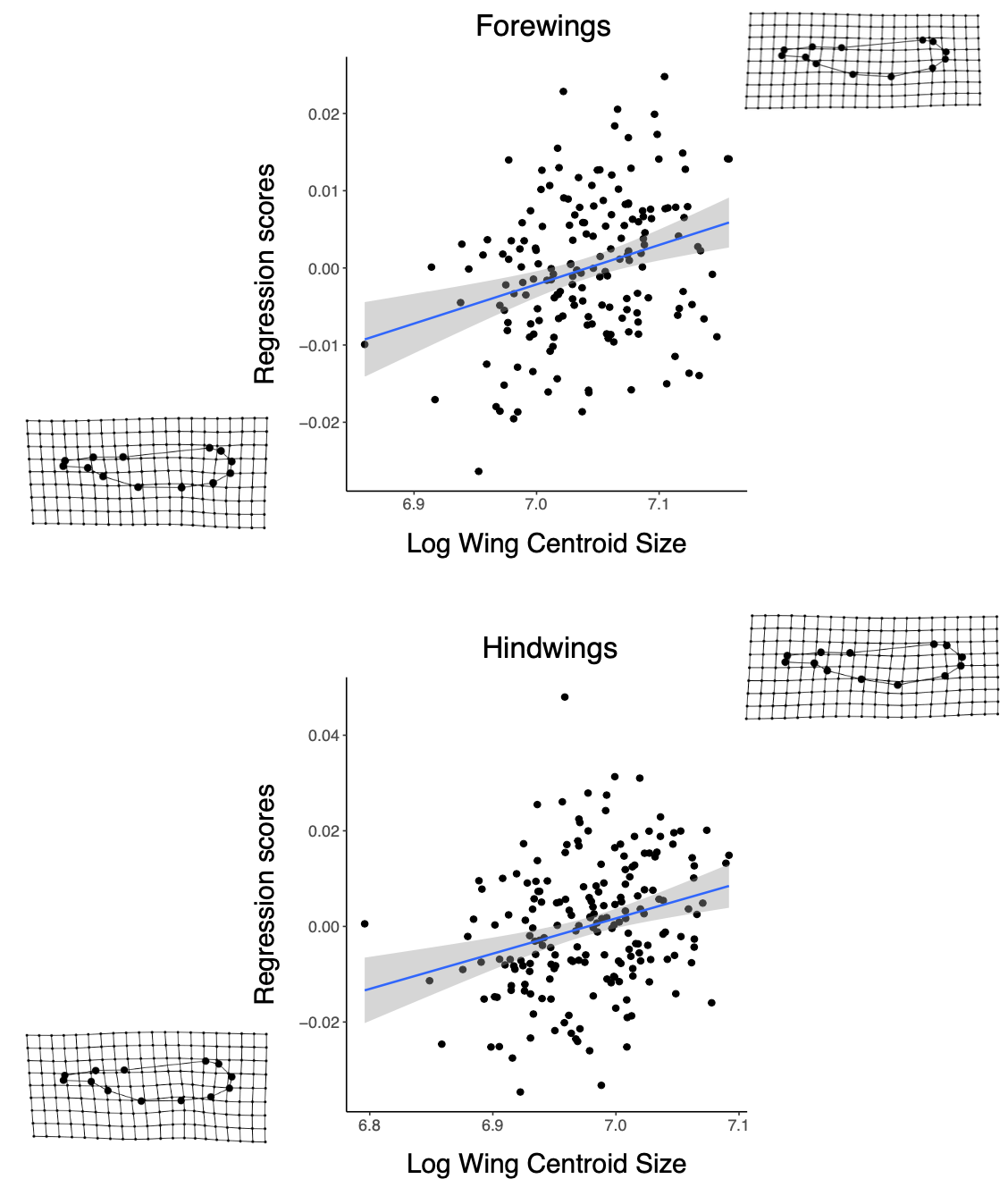


Figure S9. Variation of wing shape (depicted as regression scores) along the variation of wing centroid size for fore- (top) and hindwings (bottom). The regression line shows a linear fit, and the shaded region represents the 95% CI around the line. The deformation grids show the variation of wing shape at the most top right and most bottom left individuals on the plot. Deformation grids are magnified x20 times.
