# Supplementary material for: Phylogeography and phenotypic wing shape variation in a damselfly across populations in Europe
Source: BMC Ecol Evol. 2024 Feb 3;24:19. doi: 10.1186/s12862-024-02207-4 (PMC10838002; doi:10.1186/s12862-024-02207-4)
Supplement: Supplementary file 8 — Additional file 8. [file 12862_2024_2207_MOESM8_ESM.docx]

Table S7. Pairwise comparisons of population differences in wing shape estimated as distances (d) between populations least squares means.

|  | **Forewings** | | | **Hindwings** | | |
| --- | --- | --- | --- | --- | --- | --- |
| **Populations** | d | *Z* | *P* (>d) | d | Z | *P* (>d) |
| BEL-FRA | 0.012 | -0.437 | 0.672 | 0.012 | -0.399 | 0.640 |
| BEL-GER | 0.011 | -0.650 | 0.730 | 0.014 | -0.366 | 0.636 |
| BEL-FIN1 | 0.012 | -0.367 | 0.658 | 0.012 | -0.421 | 0.673 |
| BEL-SWE5 | 0.012 | -0.476 | 0.685 | 0.010 | -0.521 | 0.694 |
| BEL-FIN2 | 0.010 | -0.672 | 0.740 | 0.012 | -0.481 | 0.665 |
| BEL-POL2 | 0.021 | 0.241 | 0.405 | 0.021 | 0.267 | 0.394 |
| BEL-POL1 | 0.013 | -0.572 | 0.703 | 0.009 | -1.154 | 0.885 |
| BEL-FIN3 | 0.013 | -0.104 | 0.536 | 0.013 | 0.609 | 0.269 |
| BEL-SWE1 | 0.019 | -0.375 | 0.629 | 0.017 | -0.366 | 0.637 |
| BEL-SPA | 0.014 | -0.435 | 0.668 | 0.014 | -0.485 | 0.687 |
| BEL-SWE2 | 0.020 | 0.310 | 0.379 | 0.017 | 0.405 | 0.341 |
| BEL-SWE4 | 0.014 | -0.105 | 0.546 | 0.011 | -0.640 | 0.731 |
| BEL-SWE3 | 0.014 | -0.614 | 0.729 | 0.011 | -0.484 | 0.689 |
| FRA-GER | 0.013 | -0.564 | 0.718 | 0.015 | -0.231 | 0.585 |
| FRA-FIN1 | 0.013 | 0.424 | 0.332 | 0.017 | 0.017 | 0.485 |
| FRA-SWE5 | 0.016 | 0.369 | 0.363 | 0.019 | -0.197 | 0.574 |
| FRA-FIN2 | 0.015 | -0.442 | 0.662 | 0.018 | -0.513 | 0.701 |
| FRA-POL2 | 0.020 | 0.967 | 0.166 | 0.018 | 0.927 | 0.182 |
| FRA-POL1 | 0.013 | -0.534 | 0.713 | 0.011 | -0.636 | 0.738 |
| FRA-FIN3 | 0.014 | 0.675 | 0.245 | 0.017 | 0.639 | 0.267 |
| FRA-SWE1 | 0.016 | -0.135 | 0.561 | 0.014 | -0.095 | 0.530 |
| FRA-SPA | 0.014 | 0.007 | 0.505 | 0.013 | -0.064 | 0.529 |
| FRA-SWE2 | 0.020 | 0.982 | 0.165 | 0.017 | 1.032 | 0.149 |
| FRA-SWE4 | 0.017 | 0.558 | 0.295 | 0.019 | -0.277 | 0.605 |
| FRA-SWE3 | 0.013 | -0.500 | 0.680 | 0.014 | -0.065 | 0.522 |
| GER-FIN1 | 0.010 | -0.571 | 0.734 | 0.017 | -0.399 | 0.651 |
| GER-SWE5 | 0.013 | -0.226 | 0.590 | 0.018 | -0.465 | 0.681 |
| GER-FIN2 | 0.016 | -0.473 | 0.690 | 0.022 | -0.350 | 0.629 |
| GER-POL2 | 0.014 | 0.403 | 0.355 | 0.012 | 0.045 | 0.477 |
| GER-POL1 | 0.006 | -1.886 | 0.968 | 0.010 | -0.177 | 0.547 |
| GER-FIN3 | 0.010 | -0.649 | 0.743 | 0.013 | -0.415 | 0.656 |
| GER-SWE1 | 0.013 | -0.489 | 0.681 | 0.012 | -0.677 | 0.749 |
| GER-SPA | 0.014 | -0.620 | 0.741 | 0.014 | -0.786 | 0.790 |
| GER-SWE2 | 0.015 | 0.273 | 0.390 | 0.012 | -0.590 | 0.733 |
| GER-SWE4 | 0.011 | -0.315 | 0.625 | 0.017 | -0.495 | 0.704 |
| GER-SWE3 | 0.012 | -1.175 | 0.885 | 0.013 | -0.915 | 0.823 |
| FIN1-SWE5 | 0.008 | -1.195 | 0.880 | 0.009 | -1.085 | 0.865 |
| FIN1-FIN2 | 0.016 | 0.167 | 0.428 | 0.013 | 0.151 | 0.427 |
| FIN1-POL2 | 0.013 | -0.323 | 0.629 | 0.023 | -0.169 | 0.561 |
| FIN1-POL1 | 0.011 | -0.767 | 0.774 | 0.012 | -0.180 | 0.568 |
| FIN1-FIN3 | 0.004 | -2.736 | 0.998 | 0.009 | -0.294 | 0.612 |
| FIN1-SWE1 | 0.015 | -0.627 | 0.733 | 0.022 | -0.436 | 0.664 |
| FIN1-SPA | 0.014 | -1.031 | 0.847 | 0.019 | -0.621 | 0.723 |
| FIN1-SWE2 | 0.014 | -0.247 | 0.601 | 0.019 | -0.055 | 0.535 |
| FIN1-SWE4 | 0.006 | -1.998 | 0.983 | 0.007 | -1.350 | 0.904 |
| FIN1-SWE3 | 0.011 | -0.784 | 0.793 | 0.012 | -0.497 | 0.684 |
| SWE5-FIN2 | 0.014 | -0.004 | 0.501 | 0.010 | -0.105 | 0.546 |
| SWE5-POL2 | 0.017 | -0.138 | 0.551 | 0.026 | -0.164 | 0.565 |
| SWE5-POL1 | 0.013 | -0.357 | 0.630 | 0.013 | -0.454 | 0.673 |
| SWE5-FIN3 | 0.010 | -0.973 | 0.834 | 0.013 | 0.138 | 0.447 |
| SWE5-SWE1 | 0.019 | -0.376 | 0.647 | 0.023 | -0.501 | 0.690 |
| SWE5-SPA | 0.015 | -0.577 | 0.707 | 0.020 | -0.675 | 0.743 |
| SWE5-SWE2 | 0.017 | -0.129 | 0.560 | 0.021 | -0.013 | 0.506 |
| SWE5-SWE4 | 0.009 | -1.086 | 0.857 | 0.006 | -1.923 | 0.974 |
| SWE5-SWE3 | 0.015 | -0.527 | 0.705 | 0.014 | -0.263 | 0.609 |
| FIN2-POL2 | 0.027 | 0.185 | 0.421 | 0.030 | 0.163 | 0.441 |
| FIN2-POL1 | 0.019 | -0.572 | 0.725 | 0.013 | -0.726 | 0.745 |
| FIN2-FIN3 | 0.018 | 0.416 | 0.349 | 0.017 | 0.861 | 0.203 |
| FIN2-SWE1 | 0.026 | -0.443 | 0.669 | 0.027 | -0.395 | 0.652 |
| FIN2-SPA | 0.022 | -0.464 | 0.690 | 0.023 | -0.480 | 0.684 |
| FIN2-SWE2 | 0.027 | 0.294 | 0.386 | 0.026 | 0.388 | 0.351 |
| FIN2-SWE4 | 0.018 | 0.069 | 0.487 | 0.010 | -0.294 | 0.622 |
| FIN2-SWE3 | 0.019 | -0.073 | 0.529 | 0.018 | 0.134 | 0.445 |
| POL2-POL1 | 0.011 | 0.325 | 0.358 | 0.018 | 0.609 | 0.286 |
| POL2-FIN3 | 0.012 | -0.692 | 0.740 | 0.017 | -0.424 | 0.667 |
| POL2-SWE1 | 0.009 | -0.613 | 0.742 | 0.009 | -0.843 | 0.803 |
| POL2-SPA | 0.014 | -0.488 | 0.687 | 0.014 | -0.281 | 0.616 |
| POL2-SWE2 | 0.008 | -1.571 | 0.944 | 0.010 | -0.951 | 0.831 |
| POL2-SWE4 | 0.012 | -0.374 | 0.646 | 0.025 | -0.193 | 0.572 |
| POL2-SWE3 | 0.015 | -0.392 | 0.650 | 0.016 | -0.311 | 0.614 |
| POL1-FIN3 | 0.010 | -1.048 | 0.849 | 0.012 | 0.625 | 0.276 |
| POL1-SWE1 | 0.010 | -0.667 | 0.746 | 0.016 | -0.074 | 0.532 |
| POL1-SPA | 0.011 | -0.746 | 0.775 | 0.015 | -0.036 | 0.509 |
| POL1-SWE2 | 0.013 | -0.043 | 0.528 | 0.016 | 0.662 | 0.250 |
| POL1-SWE4 | 0.011 | -0.402 | 0.636 | 0.011 | -0.573 | 0.692 |
| POL1-SWE3 | 0.012 | -1.490 | 0.930 | 0.012 | -0.564 | 0.710 |
| FIN3-SWE1 | 0.013 | -0.822 | 0.786 | 0.017 | -0.506 | 0.684 |
| FIN3-SPA | 0.012 | -1.578 | 0.945 | 0.014 | -0.878 | 0.800 |
| FIN3-SWE2 | 0.013 | -0.602 | 0.727 | 0.015 | -0.359 | 0.634 |
| FIN3-SWE4 | 0.005 | -2.933 | 0.998 | 0.012 | 0.133 | 0.450 |
| FIN3-SWE3 | 0.012 | -0.486 | 0.698 | 0.012 | -0.549 | 0.701 |
| SWE1-SPA | 0.013 | -0.593 | 0.741 | 0.012 | -0.677 | 0.754 |
| SWE1-SWE2 | 0.007 | -0.970 | 0.832 | 0.008 | -1.220 | 0.895 |
| SWE1-SWE4 | 0.015 | -0.381 | 0.640 | 0.024 | -0.508 | 0.690 |
| SWE1-SWE3 | 0.011 | -1.235 | 0.874 | 0.014 | -0.711 | 0.743 |
| SPA-SWE2 | 0.014 | -0.737 | 0.770 | 0.015 | -0.970 | 0.847 |
| SPA-SWE4 | 0.013 | -0.827 | 0.803 | 0.021 | -0.690 | 0.764 |
| SPA-SWE3 | 0.016 | -1.209 | 0.896 | 0.016 | -1.101 | 0.867 |
| SWE2-SWE4 | 0.013 | -0.498 | 0.690 | 0.020 | -0.008 | 0.512 |
| SWE2-SWE3 | 0.012 | -0.223 | 0.580 | 0.010 | -0.305 | 0.605 |
| SWE4-SWE3 | 0.012 | -0.787 | 0.782 | 0.013 | -0.310 | 0.633 |
