# Supplementary material for: Phylogeography and phenotypic wing shape variation in a damselfly across populations in Europe
Source: BMC Ecol Evol. 2024 Feb 3;24:19. doi: 10.1186/s12862-024-02207-4 (PMC10838002; doi:10.1186/s12862-024-02207-4)
Supplement: Supplementary file 9 — Additional file 9. [file 12862_2024_2207_MOESM9_ESM.docx]

Table S8. Pairwise Mahalanobis distances between the average wing shape of each population. The significance of those distances was tested by 1,000 permutations. The Holm’s corrected and uncorrected p-values are provided.

|  | **Forewings** | | | **Hindwings** | | |
| --- | --- | --- | --- | --- | --- | --- |
| **Populations** | Mahalanobis distance | Holm's correction | Uncorrected P-value | Mahalanobis distance | Holm's correction | Uncorrected P-value |
| FRA-BEL | 3.284 | 0.091 | 0.001 | 3.334 | 0.091 | 0.001 |
| GER-BEL | 2.777 | 0.240 | 0.006 | 3.101 | 0.091 | 0.001 |
| FIN1-BEL | 3.175 | 0.091 | 0.001 | 3.083 | 0.091 | 0.001 |
| SWE5-BEL | 3.615 | 0.091 | 0.001 | 2.711 | 0.182 | 0.001 |
| FIN2-BEL | 2.618 | 0.391 | 0.022 | 3.030 | 0.091 | 0.001 |
| POL2-BEL | 3.868 | 0.091 | 0.001 | 3.177 | 0.091 | 0.001 |
| POL1-BEL | 3.148 | 0.091 | 0.001 | 2.700 | 0.273 | 0.001 |
| FIN3-BEL | 3.576 | 0.091 | 0.001 | 3.231 | 0.091 | 0.001 |
| SWE1-BEL | 2.978 | 0.091 | 0.003 | 2.372 | 1.000 | 0.123 |
| SPA-BEL | 3.175 | 0.091 | 0.001 | 3.010 | 0.091 | 0.001 |
| SWE2-BEL | 3.181 | 0.091 | 0.001 | 2.486 | 1.000 | 0.005 |
| SWE4-BEL | 3.610 | 0.091 | 0.001 | 2.991 | 0.091 | 0.001 |
| SWE3-BEL | 3.345 | 0.091 | 0.001 | 3.019 | 0.091 | 0.001 |
| GER-FRA | 3.021 | 0.091 | 0.001 | 3.611 | 0.091 | 0.001 |
| FIN1-FRA | 2.777 | 0.091 | 0.001 | 3.427 | 0.091 | 0.001 |
| SWE5-FRA | 2.998 | 0.091 | 0.001 | 3.709 | 0.091 | 0.001 |
| FIN2-FRA | 2.893 | 0.091 | 0.001 | 3.551 | 0.091 | 0.001 |
| POL2-FRA | 3.007 | 0.091 | 0.001 | 3.154 | 0.091 | 0.001 |
| POL1-FRA | 2.949 | 0.091 | 0.001 | 3.051 | 0.091 | 0.001 |
| FIN3-FRA | 3.250 | 0.091 | 0.001 | 4.006 | 0.091 | 0.001 |
| SWE1-FRA | 3.597 | 0.091 | 0.001 | 3.997 | 0.091 | 0.001 |
| SPA-FRA | 3.248 | 0.091 | 0.001 | 3.165 | 0.091 | 0.001 |
| SWE2-FRA | 3.997 | 0.091 | 0.001 | 4.253 | 0.091 | 0.001 |
| SWE4-FRA | 3.516 | 0.091 | 0.001 | 3.469 | 0.091 | 0.001 |
| SWE3-FRA | 3.519 | 0.091 | 0.001 | 3.624 | 0.091 | 0.001 |
| FIN1-GER | 2.432 | 1.000 | 0.109 | 3.460 | 0.091 | 0.001 |
| SWE5-GER | 2.688 | 0.741 | 0.026 | 2.983 | 0.091 | 0.001 |
| FIN2-GER | 2.442 | 1.000 | 0.134 | 3.745 | 0.091 | 0.001 |
| POL2-GER | 2.810 | 0.091 | 0.001 | 2.787 | 0.455 | 0.010 |
| POL1-GER | 2.355 | 1.000 | 0.152 | 3.023 | 0.091 | 0.003 |
| FIN3-GER | 2.914 | 0.175 | 0.001 | 3.170 | 0.091 | 0.001 |
| SWE1-GER | 3.008 | 0.217 | 0.004 | 3.462 | 0.091 | 0.001 |
| SPA-GER | 4.119 | 0.091 | 0.001 | 4.269 | 0.091 | 0.001 |
| SWE2-GER | 3.248 | 0.091 | 0.001 | 3.098 | 0.091 | 0.001 |
| SWE4-GER | 3.034 | 0.480 | 0.030 | 2.961 | 0.091 | 0.001 |
| SWE3-GER | 2.898 | 0.308 | 0.006 | 3.347 | 0.091 | 0.001 |
| SWE5-FIN1 | 2.524 | 0.854 | 0.049 | 3.112 | 0.091 | 0.001 |
| FIN2-FIN1 | 2.314 | 1.000 | 0.181 | 2.949 | 0.091 | 0.001 |
| POL2-FIN1 | 2.338 | 0.799 | 0.049 | 2.935 | 0.091 | 0.001 |
| POL1-FIN1 | 2.737 | 0.175 | 0.011 | 2.596 | 0.637 | 0.004 |
| FIN3-FIN1 | 1.594 | 1.000 | 0.906 | 2.639 | 0.455 | 0.007 |
| SWE1-FIN1 | 3.186 | 0.091 | 0.001 | 3.523 | 0.091 | 0.001 |
| SPA-FIN1 | 3.949 | 0.091 | 0.001 | 4.166 | 0.091 | 0.001 |
| SWE2-FIN1 | 2.923 | 0.091 | 0.004 | 3.050 | 0.091 | 0.001 |
| SWE4-FIN1 | 2.491 | 1.000 | 0.247 | 2.387 | 1.000 | 0.095 |
| SWE3-FIN1 | 2.787 | 0.308 | 0.009 | 2.962 | 0.364 | 0.006 |
| FIN2-SWE5 | 2.588 | 0.832 | 0.034 | 2.764 | 0.091 | 0.002 |
| POL2-SWE5 | 2.580 | 0.391 | 0.007 | 3.502 | 0.091 | 0.001 |
| POL1-SWE5 | 2.680 | 0.441 | 0.015 | 3.222 | 0.091 | 0.001 |
| FIN3-SWE5 | 2.959 | 0.091 | 0.001 | 3.153 | 0.091 | 0.001 |
| SWE1-SWE5 | 2.950 | 0.312 | 0.002 | 3.110 | 0.091 | 0.001 |
| SPA-SWE5 | 3.526 | 0.091 | 0.001 | 4.130 | 0.091 | 0.001 |
| SWE2-SWE5 | 3.279 | 0.091 | 0.001 | 2.632 | 0.182 | 0.005 |
| SWE4-SWE5 | 2.858 | 0.832 | 0.064 | 2.288 | 1.000 | 0.121 |
| SWE3-SWE5 | 3.557 | 0.091 | 0.001 | 2.858 | 0.546 | 0.006 |
| POL2-FIN2 | 3.135 | 0.091 | 0.001 | 3.489 | 0.091 | 0.001 |
| POL1-FIN2 | 2.424 | 1.000 | 0.084 | 2.866 | 0.091 | 0.001 |
| FIN3-FIN2 | 2.411 | 0.854 | 0.087 | 2.809 | 0.182 | 0.001 |
| SWE1-FIN2 | 3.222 | 0.091 | 0.001 | 3.374 | 0.091 | 0.001 |
| SPA-FIN2 | 4.027 | 0.091 | 0.001 | 4.248 | 0.091 | 0.001 |
| SWE2-FIN2 | 3.432 | 0.091 | 0.001 | 3.512 | 0.091 | 0.001 |
| SWE4-FIN2 | 2.607 | 1.000 | 0.182 | 2.341 | 1.000 | 0.098 |
| SWE3-FIN2 | 2.767 | 0.336 | 0.029 | 2.972 | 0.182 | 0.004 |
| POL1-POL2 | 2.093 | 1.000 | 0.188 | 1.965 | 1.000 | 0.397 |
| FIN3-POL2 | 2.742 | 0.091 | 0.001 | 2.969 | 0.091 | 0.001 |
| SWE1-POL2 | 2.798 | 0.148 | 0.004 | 2.945 | 0.637 | 0.006 |
| SPA-POL2 | 3.898 | 0.091 | 0.001 | 3.234 | 0.091 | 0.001 |
| SWE2-POL2 | 3.180 | 0.091 | 0.001 | 3.146 | 0.091 | 0.001 |
| SWE4-POL2 | 3.302 | 0.091 | 0.002 | 2.873 | 0.182 | 0.002 |
| SWE3-POL2 | 3.474 | 0.091 | 0.001 | 3.392 | 0.091 | 0.001 |
| FIN3-POL1 | 2.952 | 0.091 | 0.001 | 3.088 | 0.091 | 0.001 |
| SWE1-POL1 | 2.758 | 0.741 | 0.017 | 3.176 | 0.091 | 0.003 |
| SPA-POL1 | 3.679 | 0.091 | 0.001 | 3.320 | 0.091 | 0.001 |
| SWE2-POL1 | 3.485 | 0.091 | 0.001 | 3.165 | 0.091 | 0.001 |
| SWE4-POL1 | 3.020 | 0.325 | 0.016 | 2.620 | 1.000 | 0.014 |
| SWE3-POL1 | 3.412 | 0.091 | 0.001 | 3.365 | 0.091 | 0.001 |
| SWE1-FIN3 | 3.400 | 0.091 | 0.001 | 3.572 | 0.091 | 0.001 |
| SPA-FIN3 | 4.401 | 0.091 | 0.001 | 4.141 | 0.091 | 0.001 |
| SWE2-FIN3 | 3.701 | 0.091 | 0.001 | 3.560 | 0.091 | 0.001 |
| SWE4-FIN3 | 2.376 | 1.000 | 0.331 | 2.361 | 1.000 | 0.091 |
| SWE3-FIN3 | 2.852 | 0.175 | 0.003 | 3.858 | 0.091 | 0.001 |
| SPA-SWE1 | 3.594 | 0.091 | 0.001 | 3.225 | 0.091 | 0.001 |
| SWE2-SWE1 | 2.350 | 1.000 | 0.331 | 2.130 | 1.000 | 0.472 |
| SWE4-SWE1 | 3.341 | 0.148 | 0.003 | 3.612 | 0.091 | 0.001 |
| SWE3-SWE1 | 3.171 | 0.091 | 0.002 | 3.259 | 0.182 | 0.004 |
| SWE2-SPA | 4.163 | 0.091 | 0.001 | 4.183 | 0.091 | 0.001 |
| SWE4-SPA | 4.363 | 0.091 | 0.001 | 4.154 | 0.091 | 0.001 |
| SWE3-SPA | 4.733 | 0.091 | 0.001 | 4.586 | 0.091 | 0.001 |
| SWE4-SWE2 | 3.199 | 0.192 | 0.011 | 2.949 | 0.182 | 0.001 |
| SWE3-SWE2 | 2.963 | 0.290 | 0.008 | 2.459 | 1.000 | 0.085 |
| SWE3-SWE4 | 2.815 | 1.000 | 0.084 | 2.643 | 1.000 | 0.052 |
